# Isolation and characterization of a bioactive compound from *Sphingomonas sanguinis* DM with cytotoxic and molecular docking analysis

**DOI:** 10.1038/s41598-025-99178-3

**Published:** 2025-05-08

**Authors:** Mohamed A. Awad, Hesham S. M. Soliman, Samir F. El-Mashtoly, Yara E. Mansour, Bahig El-Deeb, Sherif F. Hammad

**Affiliations:** 1 Biotechnology Program, Institute of Basic and Applied Science, Egypt-Japan University of Science and Technology (E-JUST), New Borg El-Arab City, 21934 Alexandria Egypt; 2 Botany and Microbiology Department, Faculty of Science, Sohag University, Sohag, 82524 Egypt; 3 Department of Pharmacognosy, Faculty of Pharmacy, Helwan University, Ain-Helwan, Cairo, 11795 Egypt; 4 Department of Pharmaceutical Chemistry, Faculty of Pharmacy, Helwan University, Ain-Helwan, Cairo, 11795 Egypt; 5 PharmD Program, Egypt-Japan University of Science and Technology (E-JUST), New Borg El-Arab City, 21934 Alexandria Egypt; 6 Leibniz Institute of Photonic Technology, Albert-Einstein-Straße, 07745 Jena, Germany; 7 Pharmaceutical Organic Chemistry Department, Faculty of Pharmacy, Helwan University, Ain-Helwan, Cairo, 11795 Egypt

**Keywords:** *Datura metel*, *Sphingomonas sanguinis*, Nonribosomal peptide synthetase, Iturin, Skin cancer, Apoptosis, Biotechnology, Microbiology

## Abstract

*Datura metel*, a common plant in the Solanaceae family, is known for its valuable medicinal properties. The metabolites created by its rhizosphere bacterium, *Sphingomonas sanguinis* DM, have garnered interest for their potential biological effects. This study will discuss the steps involved in fermenting and processing a bacterial strain to extract potent secondary metabolites. The ethyl acetate extract of the propagated strain was subjected to fractionation and purification through various chromatographic techniques. The purified compound was characterized through multiple spectroscopic methods for structure elucidation, including UV, MS, 1D, and 2D-NMR. Its cytotoxic activity was assessed on malignant skin cells (A-431) using the MTT test compared with normal melanocytes (HFB 4). Furthermore, A-431 cells were double-stained with PI and annexin V-FITC and analyzed by flow cytometry to detect Apoptosis. Molecular investigations include PCR screening to detect genes related to the biosynthesis of bioactive metabolites, such as NRPS and lipopeptide *ItuD* genes. A prospective effective strategy to overcome tumor plasticity in melanoma is to target the Wnt signaling pathways. Molecular docking studies were conducted in the different proteins (Fz4-CRD, LRP6, GSK3β) of the Wnt signaling pathway and Protein Kinase B/Akt for the isolated compound to investigate the possible pathway to inhibit melanoma. *Sphingomonas sanguinis* DM produced bis (2-methylheptyl) benzene-1,4-dicarboxylate isolated for the first time from a natural source. It was cytotoxic against the A-431 human skin carcinoma cell line (IC_50_ = 191.61 µg/mL) but less effective against HFB 4 human normal melanocytes (IC_50_ = 416.23 µg/mL; selectivity index = 2.17). The A-431 cells showed a significant increase in early Apoptosis and a moderate rise in late Apoptosis. PCR amplification confirmed genes encoding A domain and Iturin A. Bacterial sequences are available in NCBI GenBank with accession codes OR597597 and OR597598. Consequently, *Sphingomonas sanguinis* DM synthesized a cytotoxic natural terephthalate diester derivative, along with the host specificity of the strain.

**Supplementary Information:**

The online version contains supplementary material available at 10.1038/s41598-025-99178-3.

## Introduction

It is crucial to ensure sufficient resources to address the growing pharmacological needs of our population^1^. One effective strategy to achieve this is through natural product research. This area has opened opportunities to discover novel chemical structures with potent and selective biological activities^2^. Natural products play a central role in initiatives to develop innovative treatments to mitigate the spread of diseases and reduce mortality rates^3^. Furthermore, the random screening of natural products has dramatically facilitated the discovery of new drugs^4^. While there is a current emphasis on synthetic products, bioactive natural compounds remain an essential element of modern medicine. In addressing the rising challenge of drug resistance in both human and plant pathogens, there is an urgent need for new alternatives. Scientific evidence demonstrates that natural compounds with cytotoxic properties can effectively target and eliminate tumor cells. Some of these compounds are currently employed in chemotherapy treatments, while others have exhibited promising potential in preclinical studies as agents capable of combating tumors and metastasis^5^.

Solanaceae family includes the *Datura* genus, which can be found in various parts of the world. *Datura metel*, *D. innoxia*, and *D. stramonium* are the most commonly used for medicinal purposes. Despite its poisonous nature, *D. metel* has been traditionally used to treat a variety of ailments, including madness, epilepsy, heart disease, and diarrhea, among others^6^. The herb known as *D. metel* is recognized for its various properties, such as astringent, bitter, germicidal, acrid, anti-phlogistic, anodyne, narcotic, antiseptic, and soothing. Different parts of this herb can be utilized for their hallucinogenic, narcotic, anti-tussive, antispasmodic, bronchodilator, and anti-asthmatic effects. They can aid in treating skin diseases, painful menstruation, hemorrhoids, wounds, burns, and skin ulcers^7^. This plant has been found to possess a wide range of biological activities, including antioxidant, anti-inflammatory, antimicrobial, insecticidal, anticancer, anti-diabetic, analgesic, antipyretic, neurological, contraceptive, and wound healing properties^6^.

The rhizosphere, a narrow soil layer surrounding and impacted by plant roots, harbors many microorganisms, notably bacteria^8^. This varied microbial population is crucial in enhancing plant growth, facilitating nutrient uptake, and protecting plant well-being^9^. Recently, a growing interest has been in identifying environmentally friendly substitutes for hazardous chemicals and pesticides^10^. For instance, *Sphingomonas* is present in several plant-based settings, including the rhizosphere^11^. It is a rod-shaped bacteria characterized by its distinct yellow pigment. It gained attention for its impressive ability to thrive in harsh conditions and break down environmental toxins and pollutants^12^. The current authors previously isolated *Sphingomonas sanguinis* DM from the rhizosphere of *Datura metel* and identified it molecularly through 16S rRNA gene sequencing^13^*.*

Researchers study microorganisms for their potential beneficial environmental roles, such as secondary metabolite production^14^. Microbes produce these metabolites due to their genetic makeup and habitat-specific symbiosis^15^. Nonribosomal peptide synthase (NRPS) is a biosynthetic system in microorganisms involved in producing various biologically active compounds. Cyclopeptides, a significant chemical family, possess structural characteristics and are created by NRPSs^16^. Previous studies have established the biosynthetic origins of natural products in symbiotic microbes due to the rapid growth of molecular genetics related to nonribosomal peptides in microorganisms^17^. Identifying the microbial biosynthetic source that produces the desired natural compound from the host organism is crucial^18^. However, metagenomic analyses have revealed many nonribosomal synthase gene clusters in microorganisms^19^.

Lipopeptides are a class of antimicrobial compounds with cyclic structure and low molecular weight. They are primarily synthesized by diverse bacterial strains^20^. Lipopeptides comprise a hydrophilic head of 7–10 amino acids and a hydrophobic fatty acid tail. There are three main groups of lipopeptides that bacterial species produce, including surfactin, iturin, and fengycin families^21^. Iturins contain seven alpha-amino acids and a single beta-amino fatty acid, with fatty-acid chains ranging from C14 to C17^22^. Previous studies have confirmed that Iturins are effective against fungi^23^. Researching the host specificity of microorganisms helps determine their secondary metabolites’ potential ecological or therapeutic significance. The efficient selection of a bioactive strain depends on identifying genes responsible for the biosynthesis of secondary metabolites. Thus, combining gene screening with bioactivity screening is a possible technique^24^.

Melanoma is a form of cancer that arises in melanocytes, cells responsible for producing pigments originating in the neuroectoderm. These cells can be found in various body parts, such as the skin, iris, and rectum. In Western countries, cutaneous melanoma is quite prevalent, and its incidence is increasing^25^. Programmed cell death, or Apoptosis, plays a crucial role in the self-destruction of melanoma cells. Both positive (apoptotic) and negative (anti-apoptotic) regulators are present in the molecular components of Apoptosis in these tumors^26^. The current task at hand is to develop effective strategies to address these defects in cell death and enhance the outlook for patients in the later stages of the disease^27^. It was found that *Sphingomonas* sp. can infiltrate and infect a variety of mammalian cell lines. After infection, these cells exhibited diverse cytopathic impacts, including vacuolation in the perinuclear zone, cytoplasmic granulation, and membrane blebbing^28^. Consequently, it is crucial to explore untapped biodiversity hotspots in the quest for new natural products that may offer therapeutic benefits^29^.

The successful synthesis of terephthalate ester derivatives has proved highly effective in producing a wide range of heterocycles with excellent yields. Furthermore, their biological properties have been thoroughly evaluated, revealing good antimicrobial and antibiofilm activity against *Staphylococcus aureus, Pseudomonas aeruginosa,* and *Escherichia coli* strains^30^. Furthermore, aromatic carboxylic acid derivatives exhibited significant anti-proliferative effects against lung cancer (A549) and leukemia (P388) cell lines^4^. Terephthalate exhibits exceptional thermally and chemical stability and is highly resistant to biological atmospheric agents. As a result, it is challenging to degrade and destroy the environment^31^.

Based on the challenges above, *Sphingomonas sanguinis* DM, a strain found in the rhizosphere of *Datura metel*, has been studied as a potential source of cytotoxic secondary metabolites. Furthermore, the study sought to establish molecular evidence of *Sphingomonas sanguinis* DM’s host specificity through NRPS and lipopeptide *ItuD* phylogenies. Molecular docking was also employed to assess the isolated compound’s binding affinity and intermolecular interactions.

## Materials and methods

### Materials

The analytical-grade *n*-hexane, ethyl acetate, dichloromethane, and methanol were obtained from El Nasr Pharmaceutical Chemicals Co, Egypt. The necessary supplies, including Dimethyl sulfoxide (DMSO), MTT, and trypan blue dye, were purchased from Sigma in St. Louis, MO, USA. Also, the fetal bovine serum, RPMI-1640, HEPES buffer solution, L-glutamine, gentamycin, and 0.25% Trypsin–EDTA were procured from Lonza in Belgium.

### Collection of bacterial strain

The authors obtained *Sphingomonas sanguinis* DM from their laboratory collection after its isolation from the rhizosphere of *Datura metel*. 16S rRNA gene sequence of bacterial strain was previously deposited in the NCBI GenBank database with the accession number PP422198^13^.

### Large-scale fermentation and working up for extraction of secondary metabolites

A potent strain was utilized to produce a bacterial suspension, which was then added to a 100 mL tryptone soy broth medium and allowed to grow for two days at 37 °C to create a seed culture. Following this, 5 mL of the seed culture was distributed into ten 1 L Erlenmeyer flasks, each containing 500 mL of broth medium, and agitated at 37 °C for two weeks to stimulate growth^32^. The bacterial cultures were sonicated 30 min after incubation to cause cell rupture. The resulting broth, comprising a volume of 5 L, was extracted through maceration in 5 L of methanol. Following filtration, the methanol extract was concentrated *in vacuo*. The concentrate was suspended in water and portioned with ethyl acetate till exhaustion. Eventually, the ethyl acetate extract was evaporated till dryness (2.5 gm) and stored for subsequent purification^33,34^.

### Isolation, purification, and identification of the secondary metabolite

The ethyl acetate fraction was subjected to column chromatography using normal phase silica gel (70–230 mesh, Merck, Germany) in a 60 × 3 cm column. The column was eluted using a gradient system consisting of DCM and MeOH, and the eluted fractions were closely monitored on TLC (DC-Alufolien, silica gel 60 F254 matrix, Merck, Germany). The common fractions were collected and dried using a rotatory evaporator under a vacuum below 40⁰C. The solvent strength methylene chloride and methanol (9.8:0.2) yielded a crude fraction that was subjected to Preparative thin layer chromatography (PTLC) using a developing system composed of *n*-hexane: ethyl acetate (9:1). PTLC is a technique used for separating and purifying larger quantities of compounds compared to analytical TLC. It processes sample volumes in the microgram to milligram range and utilizes thicker plates (0.5–2 mm) to increase adsorbent capacity and enhance separation. The quality of purification on PTLC is high as the particle size of the silica gel range is 5–8 μm. This method is widely used in organic synthesis and natural product chemistry because of its simplicity and cost-effectiveness. The isolated compound was detected under a 254 nm UV light, scratched from the plate, and extracted by a mixture of methanol and methylene chloride. The solvent was distilled off to give the pure compound.

After extraction of the desired band through PTLC, the purity of the isolated compound was verified through TLC, then detection under UV light, depending on two wavelengths (254 and 365 nm), to confirm that a single compound was separated. In addition, the TLC plate is sprayed with an anisaldehyde/sulfuric acid reagent and heated (which results in the charring of any impurities and appears as a definite spot) to detect the presence of any impurities.

The 1D and 2D-NMR spectra were recorded using the Bruker Avance DRX instrument from Rheinstetten, Germany, which operated at 400 MHz (1H NMR) and 100.40 MHz (^13^C NMR). Deuterated chloroform (CDCl_3_) was used as a solvent for the isolated compound. To carry out the ESI–MS positive ion acquisition mode, the XEVO TQD triple quadruple instrument (Waters Corporation, Milford, MA0175USA, mass spectrometer) was used. The ACQUITY UPLC-BEH C_18_ 1.7 μm—2.1 × 50 mm column was utilized with a solvent system of (A) water containing 0.1% formic acid and (B) acetonitrile, with a flow rate of 0.2 mL/min. For more characterization, a Shimadzu UV-1800 spectrophotometer was used. The Raman spectra were captured using a WITec alpha 300R Raman imaging microscope integrated with a 532 nm laser line. The laser was calibrated to deliver 30mW power and 20s accumulation time.

### Extraction and purification of DNA

A micro-centrifuge tube with a volume of 1.5 mL was used to transfer the enriched Sphingomonas sanguinis DM culture. One milliliter of the culture was transferred into the tube, and the cell suspension was centrifuged for 10 min at 14,000×*g*. After centrifugation, the resulting pellet was resuspended in 300 μL of DNase-RNase-free distilled water before subjecting it to another round of centrifugation for 5 min at 14,000×*g*. After removing the supernatant, the pellet was resuspended in 200 μL of DNase-RNase-free distilled water, followed by incubating the suspension at 100 °C for 15 min and rapidly cooling it on ice. Lastly, a final round of centrifugation for 5 min at 14,000×*g* at 4 °C was performed before utilizing five microliters of the supernatant as a DNA template for PCR^35^.

### PCR Amplification

The Taq polymerase protein is used to amplify DNA samples for polymerase chain reactions (PCRs) on a T1 Thermal Cycler from Biometra, Germany. The resulting products were assessed through 1% agarose gel electrophoresis. For molecular investigation of *Sphingomonas sanguinis* DM, the strain was screened for the malonyl-CoA transacylase *ItuD* gene responsible for producing Iturin A lipopeptide. PCR amplification of the lipopeptide gene was conducted, beginning with an initial denaturation of 5 min at 95 °C, followed by 30 cycles of denaturation (1 min at 94 °C), annealing (1 min at 61 °C), extension (1 min at 72 °C), and a final extension at 72 °C for 10 min. To amplify approximately 1000 bp A regions, degenerate oligonucleotide primers NPRSF and NRPSR were utilized in a PCR reaction for detection of gene encoding nonribosomal peptide synthase (NRPS) enzyme, and followed this procedure: 3 min at 94 °C, 35 cycles at 94 °C for 1 min, 54 °C for 1 min, and 72 °C for 2 min, followed by 7 min at 72 °C^23,24^. The primers utilized for the respective genes have been listed in Table 1.

**Table 1:** PCR primers for amplification of lipopeptide *ItuD* and NRPS genes.

Target	Primers	Sequences (5′–3′)	Amplicon size (bp)
*ItuD* gene	ItuD1F	GATGCGATCTCCTTGGATGT	647
	ItuD1R	ATCGTCATGTGCTGCTTGAG	
A domain	NRPSF	GCNGGYGGYGCNTAYGTNCC	1000
	NRPSR	CCNCGDATYTTNACYTG	

### DNA sequencing

The PCR product was purified from a 1% agarose gel using a GeneDireX gel extraction kit (Taiwan). Following this, sequencing was performed by the Macrogen facility in Korea using the Big TriDye sequencing kit (ABI Applied Biosystems) with the ItuD1F and NPRSF forward primers^24^.

### Sequence analysis and GenBank accession numbers

BioEdit version 5.0.7 translated the nucleotide sequences for NRPS and ItuD into peptide sequences. The BLAST tool, developed in collaboration with the National Center for Biotechnology Information (NIH, MDUSA), was used to determine the similarity percentage between nucleotide sequences and other sequences. To create a phylogenetic tree, nucleotide sequences were aligned with various sequences obtained from GenBank using Clustal X^36^. The MEGA version 3.1 was employed to construct neighbor-joining phylogenies^37^. All nucleotide sequences obtained from *Sphingomonas sanguinis* DM throughout this study have been successfully deposited in the NCBI GenBank database.

### Molecular docking

The binding orientations and interactions of a promising isolated compound were stimulated with the crystallographic structures of Frizzled-4 signalosome assembly by stabilizing cysteine-rich domain dimerization in complex with palmitoleic acid, Low-density lipoprotein receptor-related protein 6 (LRP6) in complex with sclerostin (SOST) protein, Glycogen synthase kinase 3 in complex with AR-A014418 and Pleckstrin Homology Domain of Protein Kinase B/Akt in Complex with Covalent-Allosteric AKT Inhibitor, retrieved from the Protein Data Bank (PDB code: 5UWG1, 3SOV2, 1Q5K3 and 6HHG4). Using the MOE structure preparation module, the protein’s energy was reduced and protonated in three dimensions. Before docking, ligand and water molecules were eliminated from the crystal structure. A rigid receptor was used as the docking methodology, and the triangle matcher was used for placement to identify the docking site and dock the database of all the investigated compounds. The rescoring functions London dG and GBVI/WSA dG were selected, and a force field was introduced to facilitate further refinement. The best-scoring conformation for each molecule was determined by minimizing the free binding energy (kcal/mol). The most probable binding conformation is the bound conformation of the ligand within the binding site, with the highest docking score ^38–41^.

### Cytotoxicity

#### Mammalian cell lines

The human normal melanocytes cell line, HFB 4 cells, and the human skin carcinoma cell line, A-431 cells, were sourced from the American Type Culture Collection (ATCC) in Rockville, MD.

#### Cell line propagation

The cells were cultured using RPMI-1640 medium with 10% fetal calf serum and 50 µg/ml gentamycin and were consistently maintained at 37 °C in a humidified environment with 5% CO_2_. They were subcultured two to three times per week to ensure optimal growth and viability.

#### Cytotoxicity evaluation using viability assay

For antitumor assays, Corning® 96-well tissue culture plates were utilized to add tumor cell lines with a concentration of 5 × 10^4^ cells per well. Post a 24-h incubation period. The tested compound was introduced into the 96-well plates, with each well consisting of three replicates and varying concentrations ranging from 0 to 2000 µg/mL as specified in the Additional file. Each 96-well plate included six vehicle controls with media or 0.5% DMSO to ensure quality control. After the 48-h incubation period, the MTT test was conducted to evaluate cell viability. The media was then replaced with 100 µL of fresh RPMI 1640 culture medium free of phenol red, followed by adding 10 µL of 12 mM MTT stock solution to each well, including the untreated controls. This solution contained 5 mg of MTT in 1 mL of PBS. 96-well plates were incubated at 37 °C with a 5% concentration of CO_2_ for four hours. Following this, 85 µL of media were removed from the wells, and 50 µL of DMSO was added to each well, mixed thoroughly with the pipette, and incubated at 37 °C for 10 min. A microplate reader (SunRise, TECAN, Inc, USA) was utilized to measure the optical density at 590 nm to determine the viable cell count. Then, the formula [(ODt/ODc)] × 100% was employed to calculate the viability percentage of tumor cells. In this equation, ODt represents the average optical density of wells treated with the tested sample, while ODc represents the average optical density of untreated cells. The survival curve of each tumor cell line after treatment with the specified compound is obtained by plotting the relation between surviving cells and drug concentration. The 50% inhibitory concentration (IC_50_) was estimated by analyzing the dose–response curve for each concentration, which represents the concentration required to cause toxic effects in 50% of intact cells^42^.

#### Apoptosis detection

The Miltenyi Biotech Annexin V-FITC apoptosis detection kit was utilized to assess Apoptosis. Cells were first seeded at a density of 3.5 × 10^5^ in a T-25 flask and allowed to incubate overnight at 37 °C in 5% CO_2_. The following day, the cells were exposed to the specified drug doses or the vehicle control. After 48-h incubation, both floating and attached cells were gathered through trypsinization and centrifugation at 2000 rpm for 5 min. This was performed to evaluate the strength of the signal. After discarding the supernatants, the cell pellets underwent two washes with 1X PBS and one wash with 1X binding buffer, followed by resuspension in 1 mL 1X binding buffer. In a new tube, 100 µL of the resuspended cells were combined with 5 μL of annexin V-FITC and 5 μL of PI. The tubes were kept in the dark at room temperature for 15 min. Lastly, 400 μL of 1X binding buffer was added, and the stained cells were examined using a flow cytometer (BD FACS Calibur, BD Biosciences)^43^ at the Flow Cytometry Service core facility at the Center of Excellence for Research in Regenerative Medicine and its Applications, Alexandria University, Egypt.

### Statistical analysis

The in vitro anticancer cell proliferation screening assays involved testing the indicated compound concentrations twice in triplicate. All graphs and statistical analysis, including IC_50,_ were done using GraphPad Prism software (Version 8, San Diego, CA, USA). To compare the mean cell proliferation at indicated concentrations with the control cells treated with DMSO, the student’s t-test (two-tailed) was used. The conditions that have significant differences are represented by ‘*’ (*p*-value ≤ 0.05), ‘**’ (p-value ≤ 0.01), ‘***’ (p-value ≤ 0.001) and ‘****’ (p-value ≤ 0.0001).

## Results

### Structure elucidation of the isolated compound

**Fig. 1:**
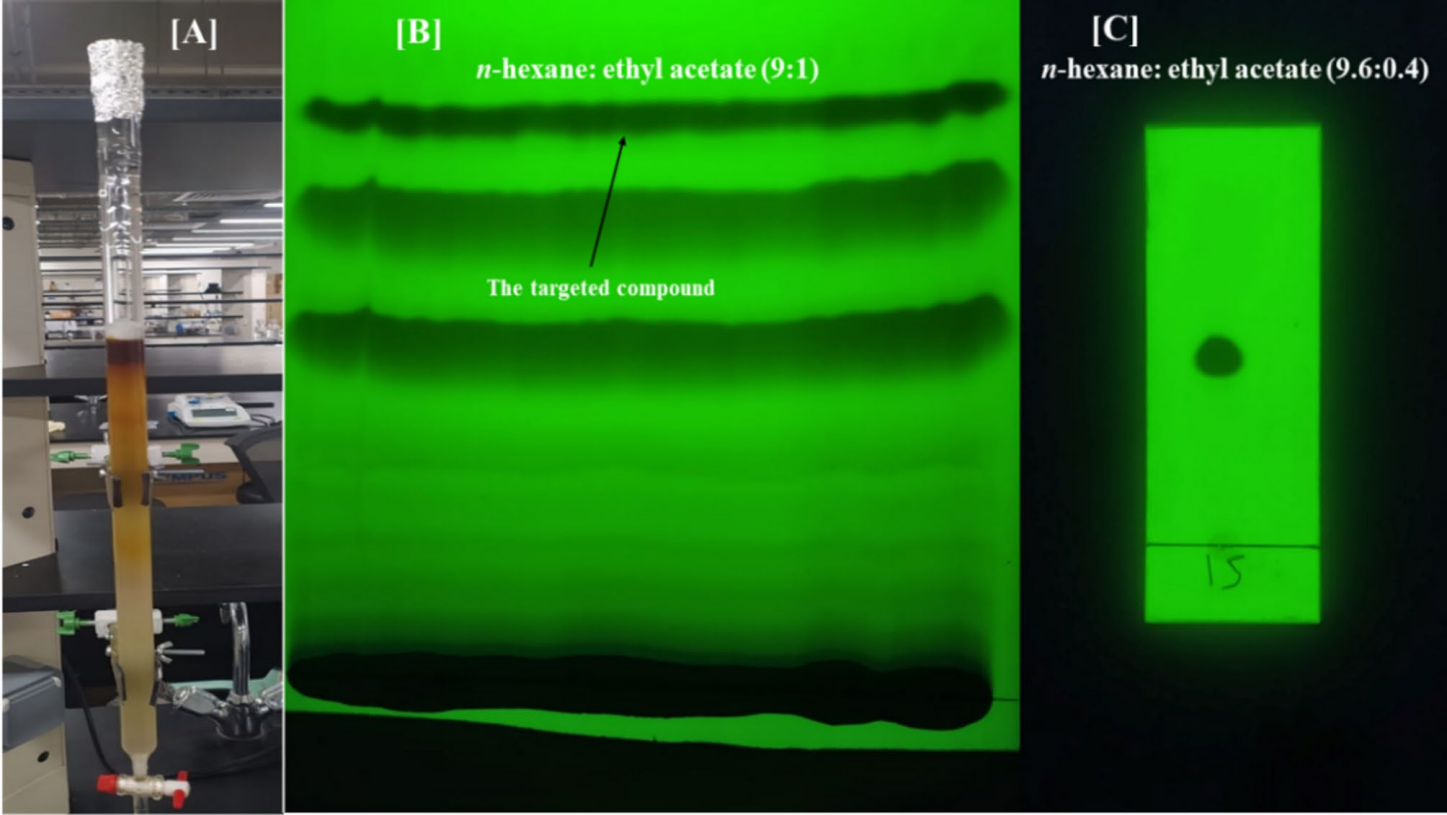
Various chromatography methods were employed to isolate and purify the isolated compound. (**A**) The total crude extract undergoes column chromatography, (**B**) Preparative TLC. [**C**] TLC of the isolated compound under UV light (254 nm).

After subjecting the bacterial crude extract to fractionation and purification through various chromatographic techniques, the pure compound was successfully separated (Fig. 1). This compound (42.9 mg) has R_f_ = 0.47 using a solvent system composed of *n*-hexane: ethyl acetate (9.6:0.4) and visualized under short wavelength UV light. When dissolved in chloroform, the compound displayed UV absorbance λ_max_ at 241 nm. In ESI positive ion mode, the compound exhibited a particular peak at m/z = 413.3168 [M^+^ + Na], corresponding to its molecular formula C_24_H_38_O_4_. ^1^H NMR (CDCl_3_, Bruker 400 MHz): a singlet peak at δ_H_ 8.12, (H 2, 3, 5 and 6). A multiplet at δ_H_ 4.28 (H-1′, H-1″). Two multiplet at δ_H_ 0.93 and δ 0.87 (H-8′, H-8″) and (H-7′, H-7″) respectively. In addition to multiplets appear at δ_H_ 1.74 (H-2′, H-2″), δ_H_ 1.46 (H-3′, H-3″), δ_H_ 1.42 (H-4′, H-4″), δ_H_ 1.26 (H-5′, H-5″), and δ_H_ 1.27 (H-6′, H-6″). ^13^C NMR (CDCl_3_, Bruker 100 MHz): The peak at δ_C_ 165.95 (C-7, C-8), δ_C_ 134.23 (C-1 and C-4) and δ_C_ 129.47 (C-2, C-3, C-5, and C-6). The peak at δ_C_ 67.77 (C-1′, C-1″), while the rest of the carbons appear at δ_C_ 38.91 (C-2′, 2″), δ_C_ 23.98 (C-3′, C-3″), δ_C_ 30.57 (C-4′, C-4″), δ_C_ 28.98 (C-5′, C-5″), δ_C_ 22.95 (C-6′, C-6″) and the peaks at δ_C_ 14.02 (C-8′, C-8″) and δ_C_ 11.08 (C-7′, C-7″) (Additional file).

**Fig. 2:**
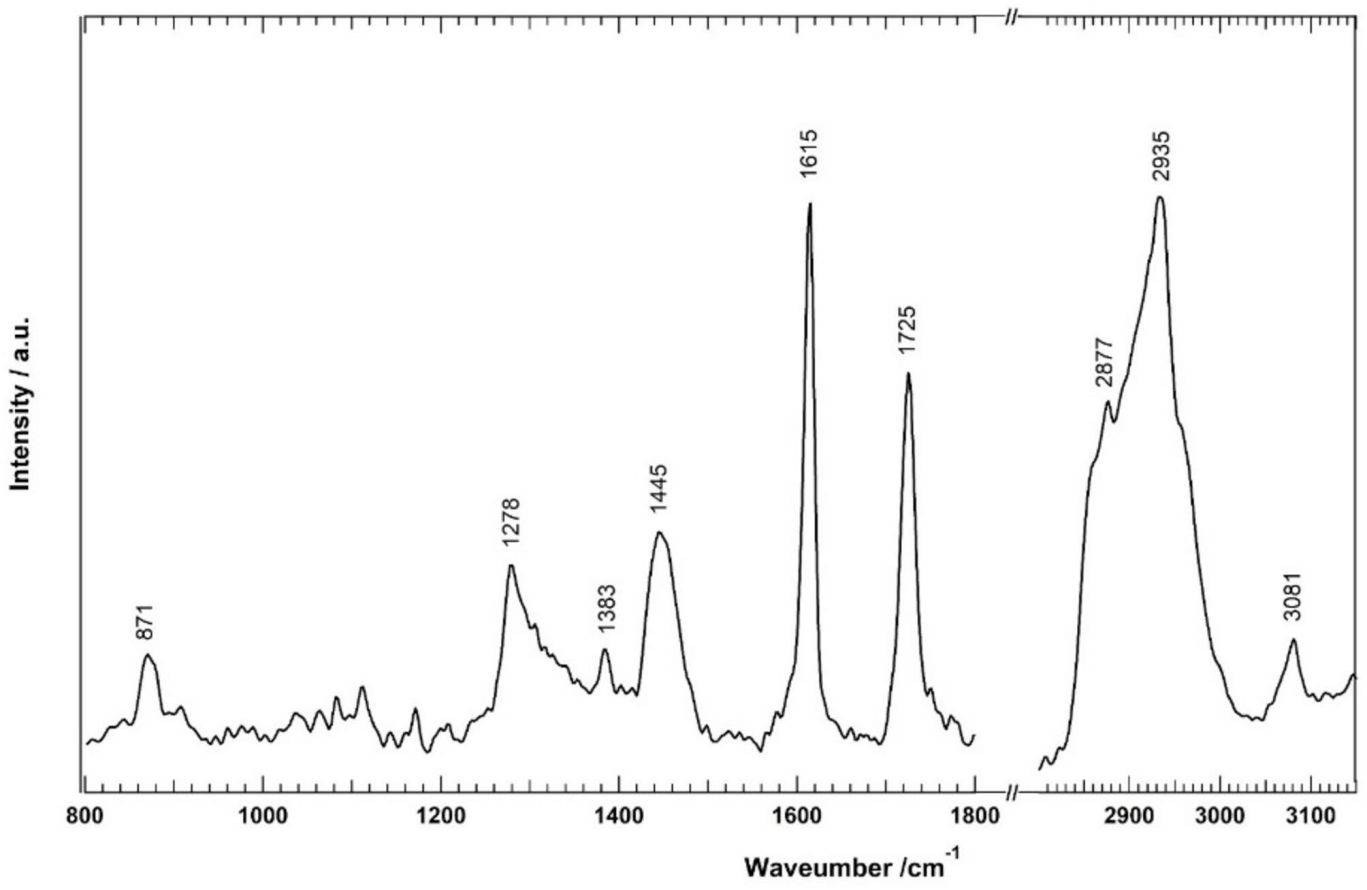
Raman spectrum of the isolated compound.

Raman spectroscopy is a powerful analytical method that can monitor the chemical composition of different specimens. This technique has been used in various fields, including material science, biomedicine, and pharmaceuticals, since it offers information regarding the chemical structure, composition, and bonding of molecules^44,45^. The Raman spectrum of this compound was measured as shown in Fig. 2. Several Raman bands were detected, and the assignment of these bands is summarized in the Additional file. The Raman bands located at 1725, 1383, and 1278 cm^−1^ are assigned to C=O stretching, COO symmetric stretching, and C–O stretching, indicating the presence of the COO group. In addition, the Phenyl-H Raman band at 3081 cm^−1^ indicates the presence of the phenyl group. The C–H stretching of CH_3_ and CH_2_ groups are observed at 2935 and 2877 cm^−1^.

### Screening of NRPS and lipopeptide *ItuD* genes

**Fig. 3:**
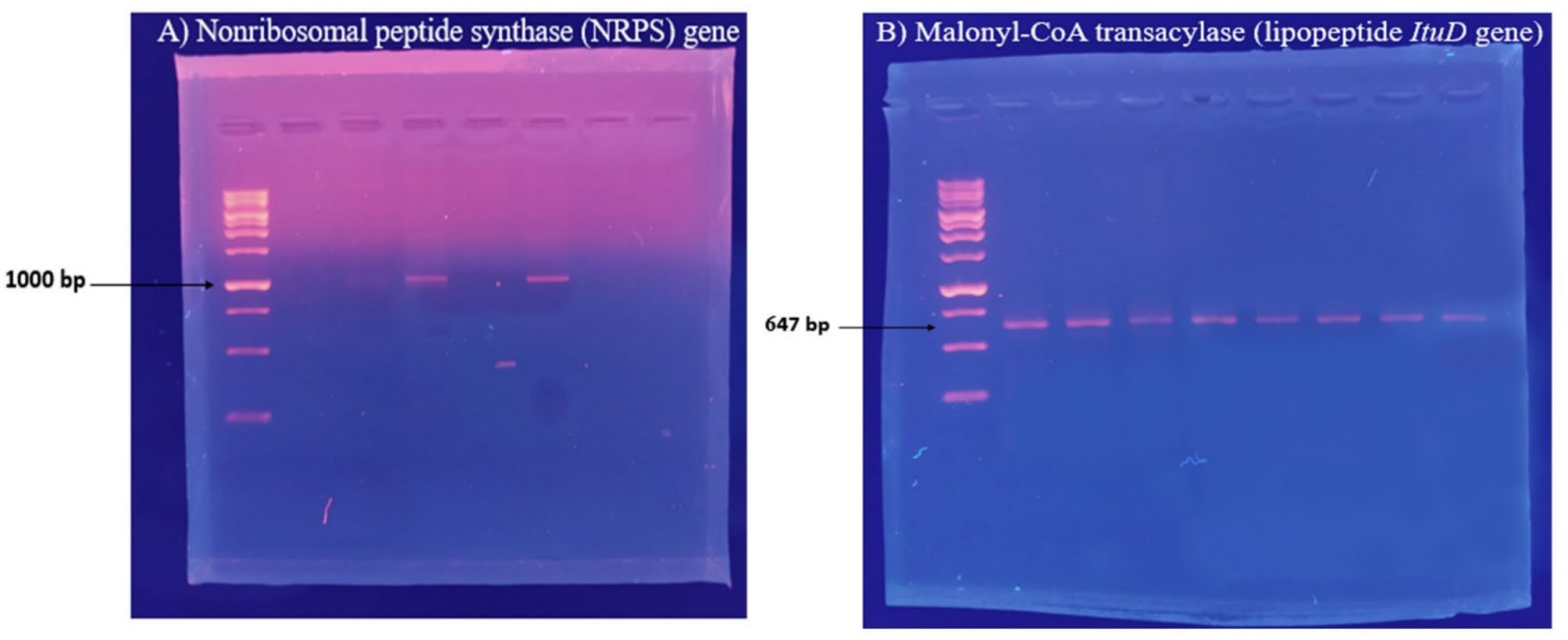
PCR amplification shows the presence of nonribosomal peptide synthase (**A**) and lipopeptide *ItuD* (**B**) genes.

According to the molecular investigation, the putative A domain of NRPS was effectively amplified from the DNA template of *Sphingomonas sanguinis* DM. The *ItuD* gene, responsible for producing the lipopeptide malonyl-CoA transacylase, was identified in the same strain via PCR amplification, as illustrated in Fig. 3, signifying its biological activity. To ensure the accuracy of the initial PCR results, gradient NRPS and ItuD PCRs were carried out utilizing various annealing temperatures. The amplified fragments underwent sequencing to verify the presence of the lipopeptide ItuD gene and NRPS A domain. All unique clones with the appropriate size (approximately 647 bp for the lipopeptide *ItuD* gene and 1000 bp for the NRPS A domain) were sequenced. A BLAST analysis was conducted in GenBank (blastx), which indicated that the NRPS A fragments from *Sphingomonas sanguinis* DM had the highest similarity to the A fragments of *Bacillus subtilis*, with a percentage identity of 66.15%.

**Fig. 4:**
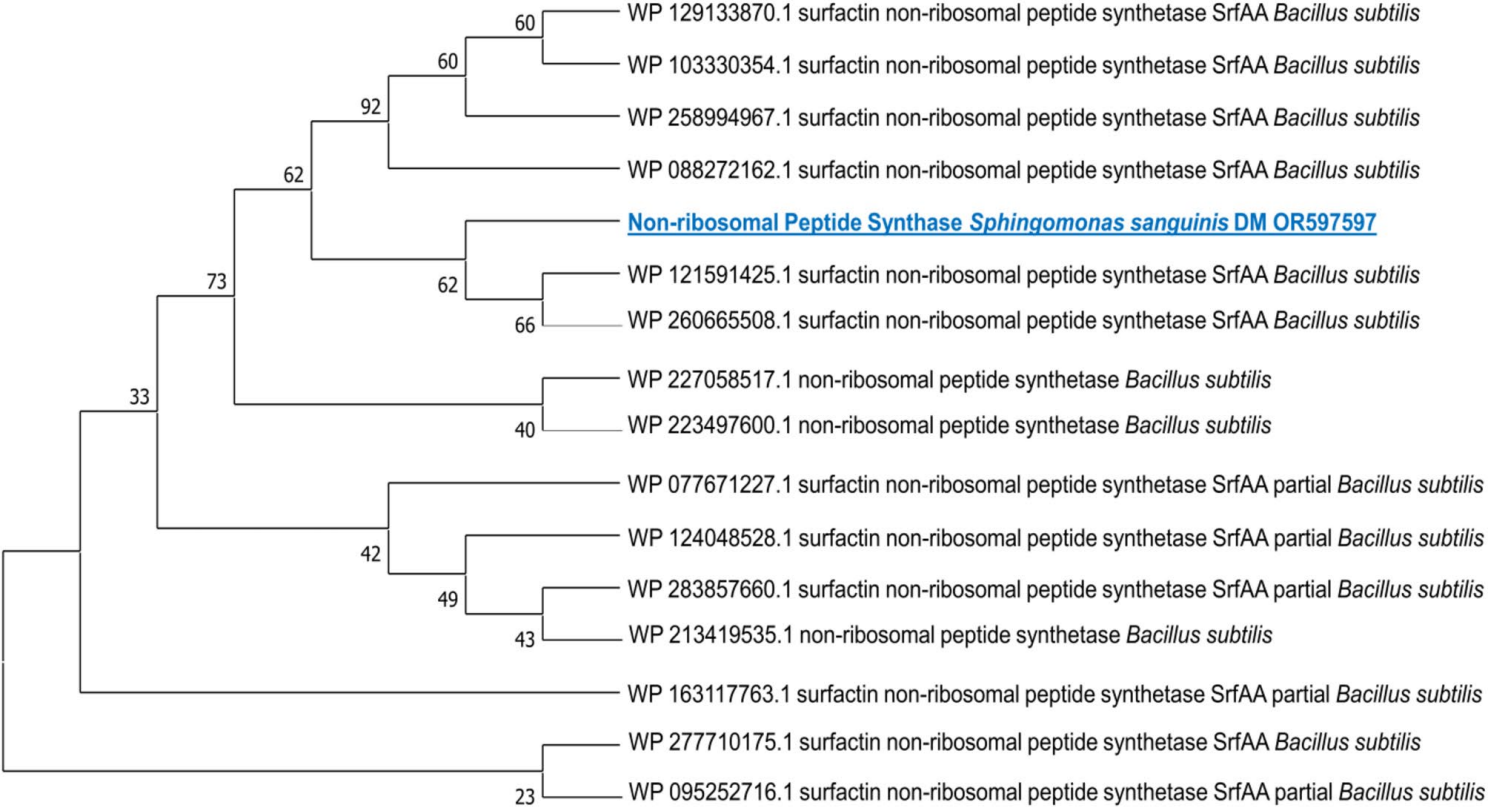
Phylogenetic analysis (1000 bootstrap replicates) of NRPS A domain from *Sphingomonas sanguinis* DM compared to other microorganisms’ diverse A fragments of *Bacillus subtilis.*

**Fig. 5:**
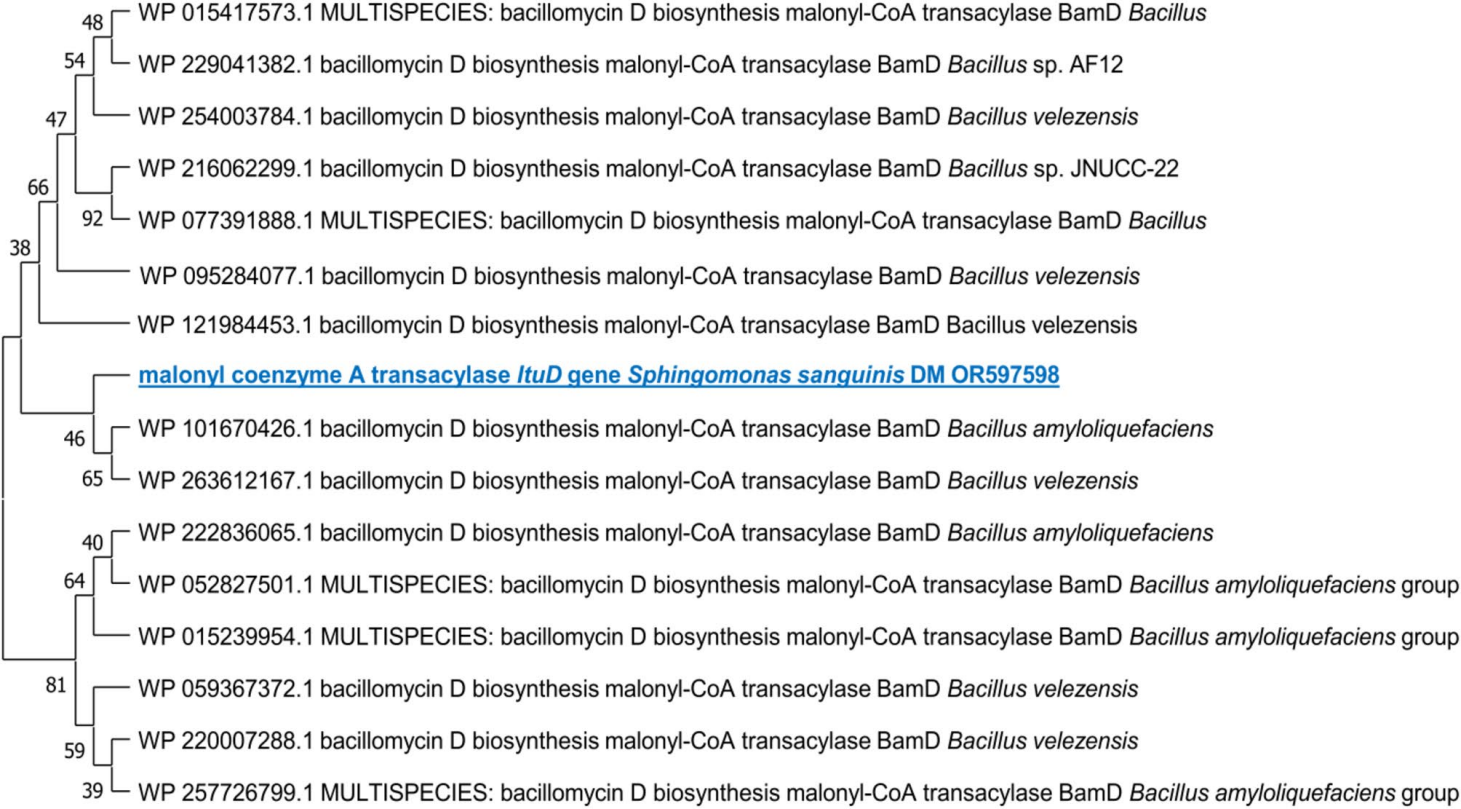
Phylogenetic analysis (1000 bootstrap replicates) of lipopeptide *ItuD* gene from *Sphingomonas sanguinis* DM compared with various lipopeptide *ItuD* fragments of *Bacillus amyloliquefaciens* and *Bacillus velezensis.*

Meanwhile, the lipopeptide *ItuD* fragments from the same strain bear resemblances to the fragments of *Bacillus amyloliquefaciens* and *Bacillus velezensis* (93% and 95%, respectively) that are already available in GenBank as detailed in the Additional file. Figure 4 and 5 illustrate phylogenetic trees that were created to compare the A domains and *ItuD* genes of various microorganisms. The bacterial sequences studied have been deposited in the NCBI GenBank database and can be accessed by the following accession numbers: OR597597 for the NRPS gene and OR597598 for the lipopeptide *ItuD* gene.

### Cytotoxicity screening

**Fig. 6:**
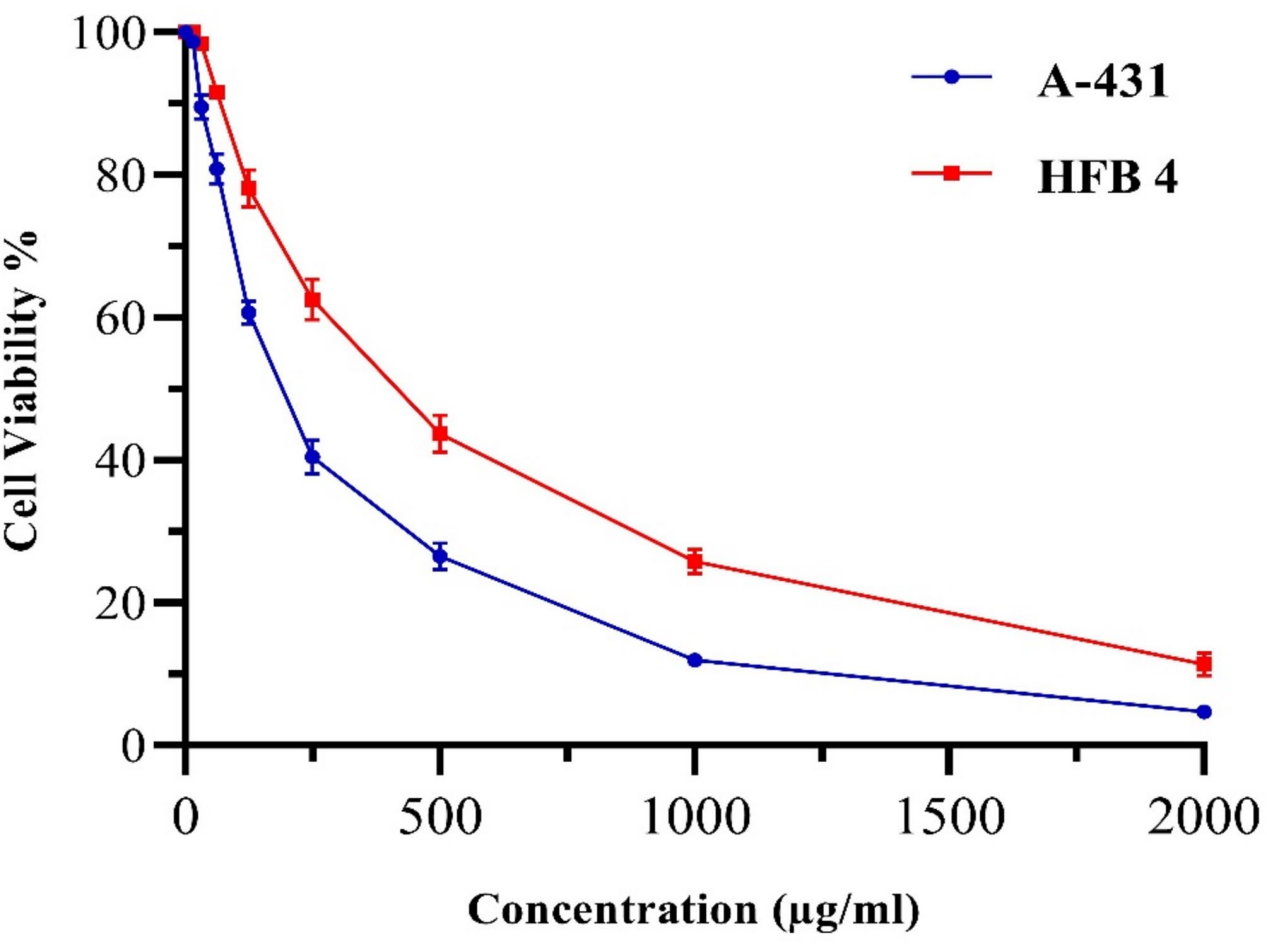
Cytotoxic activity shown as a % Cell Viability and dose–response of the isolated drug against skin carcinoma cell line (A-431) compared to normal melanocyte cell line (HFB 4) at different concentrations (0–2000 μg/mL) after treatment for 48 h. The results are presented as the mean ± S.D (n = 3).

The isolated pure compound from Sphingomonas sanguinis DM was tested for its potential anticancer properties in a laboratory setting. Specifically, its effects were evaluated on the human skin carcinoma cell line (A-431) and human normal melanocytes cell line (HFB 4) by treating the cells with various concentrations of the compound (ranging from 0 to 2000 μg/mL) for 48 h, with DMSO serving as a blank. The data in Fig. 6 illustrates the reduction process of yellow MTT to purple formazan by metabolically active cells.

According to the study, this compound demonstrated a significant cytotoxic effect against the A-431 human skin carcinoma cell line. The IC_50_ value was measured at 191.61 µg/mL, indicating that 50% of the cells remained viable. The drug exhibited gradual cytotoxic potency at 15.6 µg/mL and 500 µg/mL concentrations, resulting in approximately 98.71% and 26.53% cell viability, respectively. However, when tested against the HFB 4 human normal melanocyte cell line, the isolated compound showed the lowest cytotoxicity. The IC_50_ value was measured at 416.23 µg/mL, with cell viability of 100% and 43.70% at the same concentration, as shown in Fig. 6.

### Apoptosis detection

**Fig. 7:**
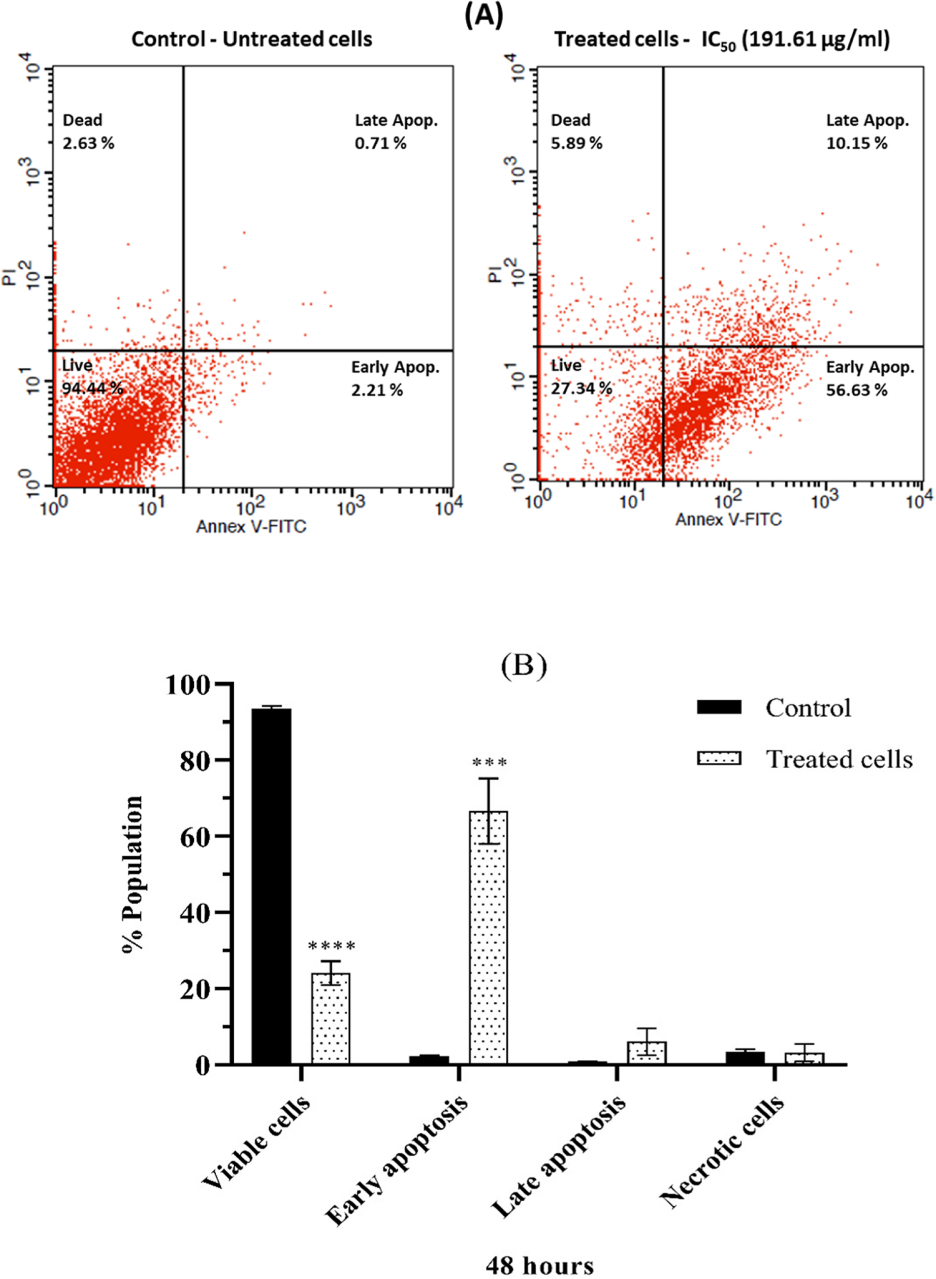
(**A**) The flow cytometric analysis of A-431 skin carcinoma cells subjected to a specific drug concentration (IC_50_ 191.61 µg/mL) versus untreated control cells after 48 h of incubation. The cells were stained with PI and Annexin V-FITC, and the percentages represent the population distribution within each quadrant, with one number chosen to represent the replicates. The dots in the lower right quadrant indicate cells in early Apoptosis, and those in the upper right quadrant represent cells in late Apoptosis. (**B**) Apoptosis detection after an incubation period of 48 h. The (*) symbol represents a significant difference compared to the control. *p < 0.05, **p < 0.01, ***p < 0.001 and ****p < 0.0001. The results are presented as the mean ± S.D (n = 3).

To determine the impact of the isolated compound on Apoptosis, the drug was administered at an IC_50_ concentration of 191.6 µg/mL to A-431 skin carcinoma cells. A comparative analysis of the treated cells was conducted with those untreated. After 48 h of incubation, the cells were double-stained with PI and annexin V-FITC, utilizing a flow cytometer for analysis. Upon exposure to the specified drug concentration, the cells showed a notable increase in early apoptotic cells at a population of 56.63% compared to the untreated control cells at 2.21% (*p* < 0.001). Furthermore, it exhibited a dramatic decrease in viable cells, reaching 27.34% (*p* < 0.0001), whereas causing a considerable rise in late apoptotic cells (10.15%) in comparison to the control (0.71%) (Fig. 7a, b; Additional file).

### Molecular docking

Table 2 provides a comprehensive overview of the specific binding amino acids and the types of bonds implicated in the interaction between the isolated compound and its corresponding target protein compared to a reference ligand.

**Table 2:** Binding amino acids in bis (2-methylheptyl) benzene-1,4-dicarboxylate and a reference ligand to the different Wnt signaling pathway proteins (Fz4-CRD, LRP6, GSK3β) and Protein Kinase B/Akt using (PDB code: 5UWG, 3SOV, 1Q5K, and 6HHG, respectively).

	PDB Id: 5UWG			PDB Id: 1Q5K		
	Binding affinity (kcal/mol)	RMSD	Interacting amino acids ∼distance (Å)	Binding affinity (kcal/mol)	RMSD	Interacting amino acids∼distance (Å)
Crystallized ligand	− 7.08	1.32	Phe135A∼4.05 Å	− 6.71	1.32	Val135 = 3.34 Å (H-bond acceptor) Val135 = 1.97 Å (H-bond donor) Ile62 ∼4 Å (hydrophobic interaction)
Terephthalate	− 8.2901	1.26	Phe137A∼3.29 Å Phe138B∼2.89 Å Pro84B∼2.80 Å	− 7.29	1.05	Val135 = 3.38 Å (H-bond donor) Val135 = 4.15 Å (H-bond acceptor) Val70∼3.4 Å (hydrophobic interaction) Lys85 = 3.26 Å (H-bond acceptor)

	PDB Id: 3SOV			PDB Id: 6HHG		
	Binding affinity (kcal/mol)	RMSD	Interacting amino acids∼distance (Å)	Binding affinity (kcal/mol)	RMSD	Interacting amino acids∼distance (Å)
Crystallized Ligand	Sclerostin (SOST) crystallized protein (no validation)			− 12.05	0.87	Asn54 = Å Trp80∼Å Val270∼Å
Terephthalate	− 5.51	1.40	Arg141 = 2.98 Å	− 9.04	0.63	Asn54 = 3.26 Å Glu85 = 3.37 Å

## Discussion

In addition to the particular peak at m/z = 413 [M^+^ + Na] which is corresponding to the M^+^ + 1 (m/z 391), the spectrum showed an important fragment at m/z 279.1190 (C_16_H_22_O_4_) due to the loss of one side chain^46^, a methyl heptyl radical (–C_8_H_17_), characteristic to benzene dicarboxylate esters.

**Fig. 8:**
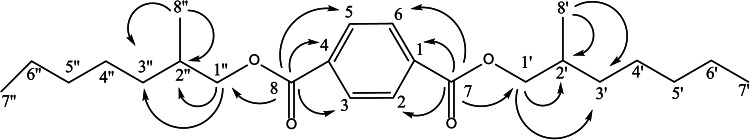
Chemical structure of a novel natural compound isolated from *Sphingomonas sanguinis* DM, showing observed key HMBC correlations.

^1^H NMR spectra of this compound showed an AA′XX′ spin system in the aromatic region with a high intensity singlet peak at δ_H_ 8.12 corresponding to protons 2, 3, 5, and 6 proving the 1,4 di-substitution of benzene ring, in addition to a multiplet at δ_H_ 4.28 corresponding to oxy-methylene groups. The two methyl groups 8′ and 8″ appear at δ_H_ 0.93, and the terminal two methyl groups 7′, 7″ appear more up-field at δ_H_ 0.87. The rest of the methylene groups appear at δ_H_ 1.74 (2′, 2″, m), δ_H_ 1.46 (3′, 3″, m), δ_H_ 1.42 (4′, 4″, m), δ_H_ 1.26 (5′, 5″, m), and δ_H_ 1.27 (6′, 6″, m). The ^13^C-NMR APT spectra revealed the presence of 11 peaks with different intensities. Each represents two identical carbons except the four identical carbons in the benzene ring which are all represented by one peak supporting the AA′XX′ spin coupling system. This was result from the symmetry of the molecule. Such carbons are: 4 quaternaries, at δ_C_ 165.95 corresponding to C-7, C-8, and at δ_C_ 134.23 corresponding to C-1 and C-4. In addition to a single, high intensity methine carbons at δ_C_ 129.47 corresponding to the four equivalent carbons C-2, C-3, C-5, and C-6 which prove the 1,4 disubstituted benzene ring. The oxy-methylene carbon appears at δ_C_ 67.77 corresponding to C-1′ and C-1″, while the rest of the methylene carbons appear at δ_C_ 38.91 (C-2′, C-2″), δ_C_ 23.98 (C-3′, C-3″), δ_C_ 30.57 (C-4′, C-4″), δ_C_ 28.98 (C-5′, C-5″), δ_C_ 22.95 (C-6′, C-6″) and the four methyl groups appear at δ_C_ 14.02 (C-8′, C-8″) and δ_C_ 11.08 (C-7′,C-7″). A useful cross peaks in HMBC between (C-8′, H-2′), (C-8″, H-2″), (C-8′, H-3′), (C-8″, H-2″), (C-8″, H-3″), (C-1′, H-2′), (C-1″, H-2″), (C-1′, H-3′), (C-1″, H-3″), (C-7, H-1′), (C-8, H-1″), (C-7, H-1, H-2, H-6, H-1′), (C-8, H-3, H-4, H-5, H-1″). Depending on the previous data, the isolated compound could be identified as bis (2-methylheptyl) benzene-1,4-dicarboxylate, as demonstrated in Fig. 8. Its synthetic origin has been described in the literature^47^.

The assignment of the Raman bands is compatible with the elucidated structure based on ^1^H and ^13^C-NMR and HMBC spectral data, where Raman bands are attributed to COO, CH_2_, CH_3,_ and Phenyl groups. To the best of our knowledge, the present study reports the first isolation and structure elucidation of this compound from a natural source.

Considerable research has focused on the application of natural products for cancer treatment. Terephthalate ester derivatives are effective in tissue engineering and controlled drug delivery systems. Furthermore, they have demonstrated a superior ability to combat Hep2 cancer cells^48^. In this regard, an intriguing discovery has been made regarding the compound bis (2-methylheptyl) benzene-1,4-dicarboxylate, which was isolated from the crude extract of a rhizosphere strain, *Sphingomonas sanguinis* DM. It has been screened for the first time and demonstrated promising cytotoxic activity as a growth inhibitor against a human skin cancer cell line. As a result, bis (2-methylheptyl) benzene-1,4-dicarboxylate could serve as a novel treatment for human skin carcinoma. In addition, research findings have indicated that an extract derived from *Sphingomonas hydrophobicum* may effectively slow down the skin’s natural aging process. This is achieved by reducing the expression of tumor suppressor proteins (p21 and p16) and increasing the expression of fibrillin-1 and versican, ultimately preventing cellular senescence. As such, it is possible that *Sphingomonas* extract could have therapeutic properties for addressing skin aging^49^.

According to the current study, most viable cells were transformed into apoptotic cells, accounting for approximately 72.66% of the population, revealing the distinct cytotoxic effect of the isolated compound. These findings are consistent with a recent study that showed many apoptotic cells across exposure groups for polyethylene terephthalate (PET)^50^. In this turn, terephthalate diester derivatives could potentially lead to long-term endoplasmic reticulum stress (ERS)-related Apoptosis or mitochondrial apoptosis^51^. Furthermore, recent studies have revealed a correlation between the Wnt/β-catenin signaling pathway and Apoptosis and the potential endocrine-disrupting effects of di(2-ethylhexyl) phthalate (DEHP)^52,53^. Another pathway, the heightened expression of glycogen synthase kinase 3-β (GSK3-β), could trigger Apoptosis by blocking prosurvival transcription factors and activating proapoptotic transcription factors^54^. In a recent study, it was found that the use of a tissue culture system with *Sphingomonas* sp. Shah resulted in the death of human lung epithelial carcinoma cells^55^. The researchers confirmed this Apoptosis through various factors, including changes in cellular morphology, nuclear marginalization, chromatin compaction condensation, a high percentage of cells with subG1 DNA content, and activation of caspase-3^55^.

To explore the hypothesis that bioactive bacteria host genes that generate secondary metabolites, degenerate oligonucleotide primers were utilized to screen the NRPS gene of a bacterial strain that had shown positive results in cytotoxic bioassays. To investigate the existence of the NRPS system in the bioactive bacterial strain, PCR primers were developed to amplify the A domains of NRPS. As illustrated in Fig. 3, the screening results verify the presence of NRPS in the bacterial strain. This study agrees with the theory demonstrating that NRPS-specific genes ought to be present in bioactive bacteria, and consequently, nonribosomal peptides were identified as the predominant structural classes for the tested strain. The current NRPS gene screening aligns with the most related study, which isolated a Gram-negative bacterial strain (*Pseudoalteromonas* sp. NJ6-3-2) from marine sponge *Hymeniacidon perleve* and confirmed the presence of A domain when screened for NRPS gene using a PCR^24^. NRPS genes are prevalent among bioactive bacterial strains, particularly within *Streptomyces*, *Pseudomonas*, and *Bacillus* genera. These genes are responsible for encoding multi-domain enzymes that synthesize secondary metabolites, which play a vital role in microbial competition and survival^9,56^. Numerous soil and marine bacteria possess NRPS pathways that enable them to produce metabolites for nutrient acquisition, competition, or defense. For example, *Streptomyces* species are prolific producers of antibiotics, including vancomycin and daptomycin^57,58^.

Hence, an NRPS gene found in a strain of bacteria may indicate a particular connection with the host. Previous studies have demonstrated that specific bacteria and their hosts share a symbiotic relationship that benefits both parties. The host supplies the bacteria with essential nutrients, such as vitamins and nutrients, while the bacteria produce specific substances, such as antibiotics, to help bolster the host’s chemical defenses^59^. To gain a deeper understanding of microbe-host interactions, it is crucial to determine the correlation between a specific bacterial strain and its host organism. Through a molecular phylogenetic analysis of the strain using its A domain, it was discovered that the A domain of *Sphingomonas sanguinis* DM bore a striking resemblance to that of *Bacillus subtilis* (as depicted in Fig. 4). Consequently, to detect the biosynthetic gene in bioactive bacteria, it is crucial to merge bioactivity screening with secondary metabolite biosynthetic gene screening. This method should involve conserved sequences from other biosynthetic pathways for PCR screening^24^.

Notably, the secretion of lipopeptide Iturin A within bacterial strains depends on the activity of a pivotal factor—the malonyl-CoA transacylase. The *ItuD* gene is responsible for encoding this factor^23^. According to previous studies, iturin boasts remarkable efficacy in combating fungal infections. Its action mechanism hinders filamentous fungi’s growth by countering sterols, phospholipids, and oleic acid in fungal membranes^21,60,61^. Additional lipopeptides, including kurstakin, maltacines, polymyxins, and surfactin-like bamylocin A, have also been identified in bacterial strains^62–65^. The genome analysis in the rhizosphere strain *Sphingomonas sanguinis* DM has verified the existence of the antifungal iturin gene, as evidenced in Fig. 3 through gene amplification screening. Such a remarkable revelation implies that *Sphingomonas sanguinis* DM could potentially discharge this antimicrobial lipopeptide in the rhizosphere of *Datura metel*, thereby safeguarding the plant from diverse phytopathogens.

Wnt signaling regulates various aspects of embryonic development, including cell fate determination, cell proliferation, cell migration, tissue organization, and organ development^66,67^. Canonical Wnt signaling activates when secreted Wnt proteins bind to the membrane co-receptors LRP5/6 (low-density lipoprotein receptor-related protein 5/6) and FZD (Frizzled). Following the activation of the disheveled protein disrupts the complex formed by adenomatous polyposis coli/axin/glycogen synthase kinase 3β. This disruption causes an accumulation of β-catenin and the building of complexes between β-catenin and transcription factors in the nucleus^66–68^. As a result, several genes downstream of this process are transcribed^69,70^. Abnormal functioning of the standard Wnt signaling pathway has been detected in various forms of cancer, especially melanoma^71^.

**Fig. 9:**
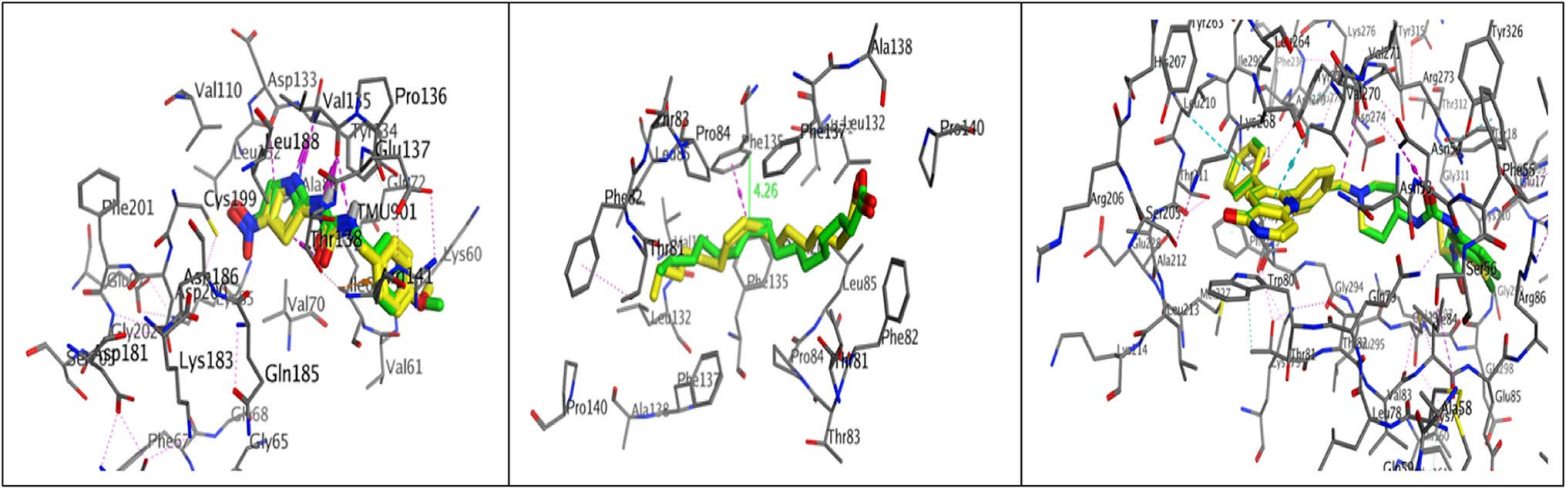
The 3D diagram illustrates the superposition of the co-crystallized ligand (green) and the redocked co-crystallized ligand (yellow) structures at the Fz4-CRD, GSK3β, and Protein Kinase B/Akt targets, respectively.

Molecular docking analyses were performed to examine the mechanisms of the natural compound bis (2-methylheptyl) benzene-1,4-dicarboxylate, which was isolated from *Sphingomonas sanguinis* DM, a bacterium found in the rhizosphere of *Datura metel*. The compound was investigated with various proteins (Fz4-CRD, LRP6, GSK3β) involved in the Wnt signaling pathway and Protein Kinase B/Akt. This was accomplished with Molecular Operating Environment software (MOE, 2014.10) to probe the complex intermolecular interactions necessary to ascertain the likely binding mode research. The MOE was employed to verify the molecular docking methods by re-docking the co-crystallized ligand of Fz4-CRD, GSK3β, and Protein Kinase B/Akt to their corresponding target proteins. This was achieved by superimposing the ligand that naturally formed a co-crystal (colored green) with the re-docked ligand and co-crystallized (colored yellow) using three-dimensional layouts. The initial configurations obtained from the PDB exhibit root mean square deviation (RMSD) values of 1.32, 1.26, and 0.87 Å, along with binding energy scores (S score) of -7.08, -6.71, and -12.05 kcal/mol, respectively (Fig. 9).

**Fig. 10:**
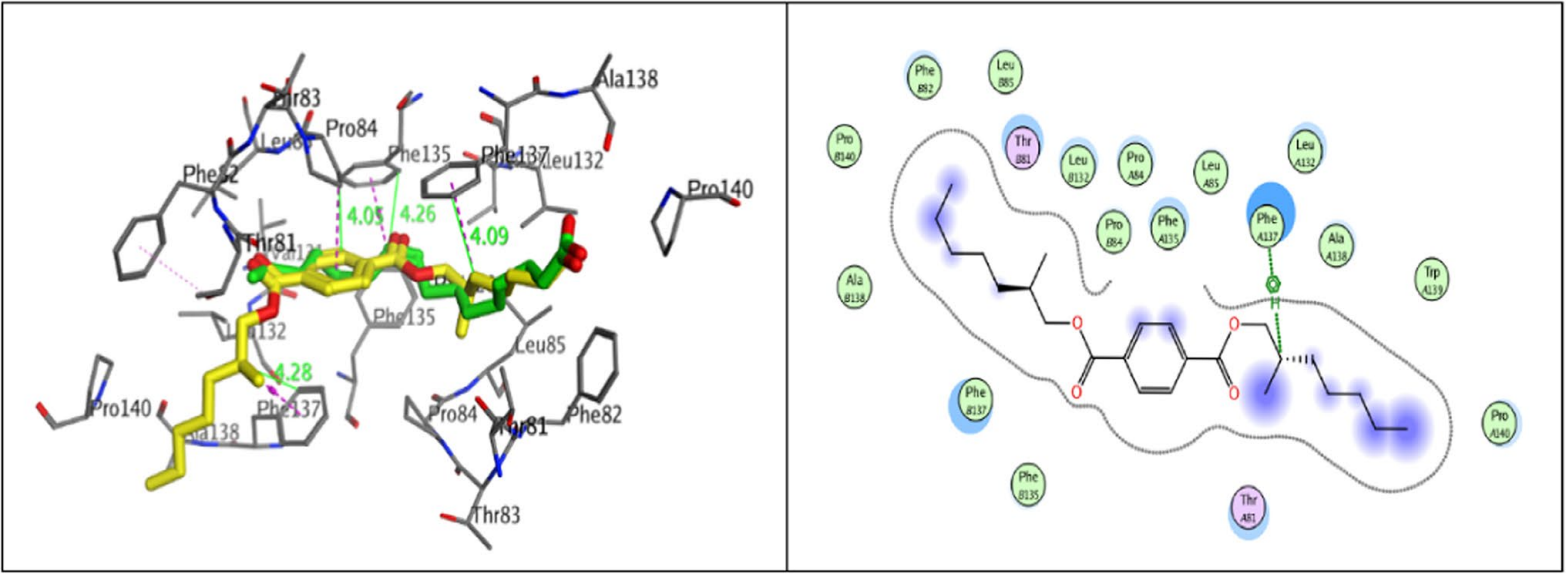
Molecular docking analysis 3D and 2D between Terephthalate with yellow color overlay the crystallized molecules with green color and Frizzled binding (PDB ID: 5UWG). (the key residues are displayed) corresponding interactions of the docked complex (Terephthalate & Frizzled).

The Frizzled receptor (FZD) is well recognized as the primary receptor for Wnt ligands. It is situated on the cell surface and interacts with Wnt ligands via a cysteine-rich domain (CRD) to enable Wnt signaling^72^. The co-crystal structure of Wnt and FZD CRD shows that the acyl chain of palmitoleic acid (crystallized molecule) on Wnt ligand is deeply buried in the hydrophobic groove of FZD CRD because multiple residues on the carboxy-terminal of Wnt bind to FZD through hydrogen bonds and hydrophobic interactions^73^. The structure reveals the requirement of mono-unsaturated fatty acid modification because the kink of the double bond on the acyl chain fits perfectly into the shape of the CRD pocket. Docking results revealed that Terephthalate can stably bind to the active pocket of FZD4-CRD with RMSD values 1.26 Å and binding energy scores of − 8.2901 kcal/mol. Additionally, the hydrophobic interactions are the most critical for the binding of Terephthalate to FZD4-CRD, as indicated by the binding mode analyses. The nonpolar side chains of numerous residues in the active sites, such as Phe82, Pro84, Leu85, Val131, Leu132, Phe135, and Phe137, are extensively contacted by the alkyl chain and benzene ring through van der Waals and hydrophobic interactions. Furthermore, the isolated molecule is stabilized in the active site by pi-pi stacking, which involves the benzene of terephthalate with residue Pro84 and the alkyl chain with residue Phe137 (Fig. 10).

**Fig. 11:**
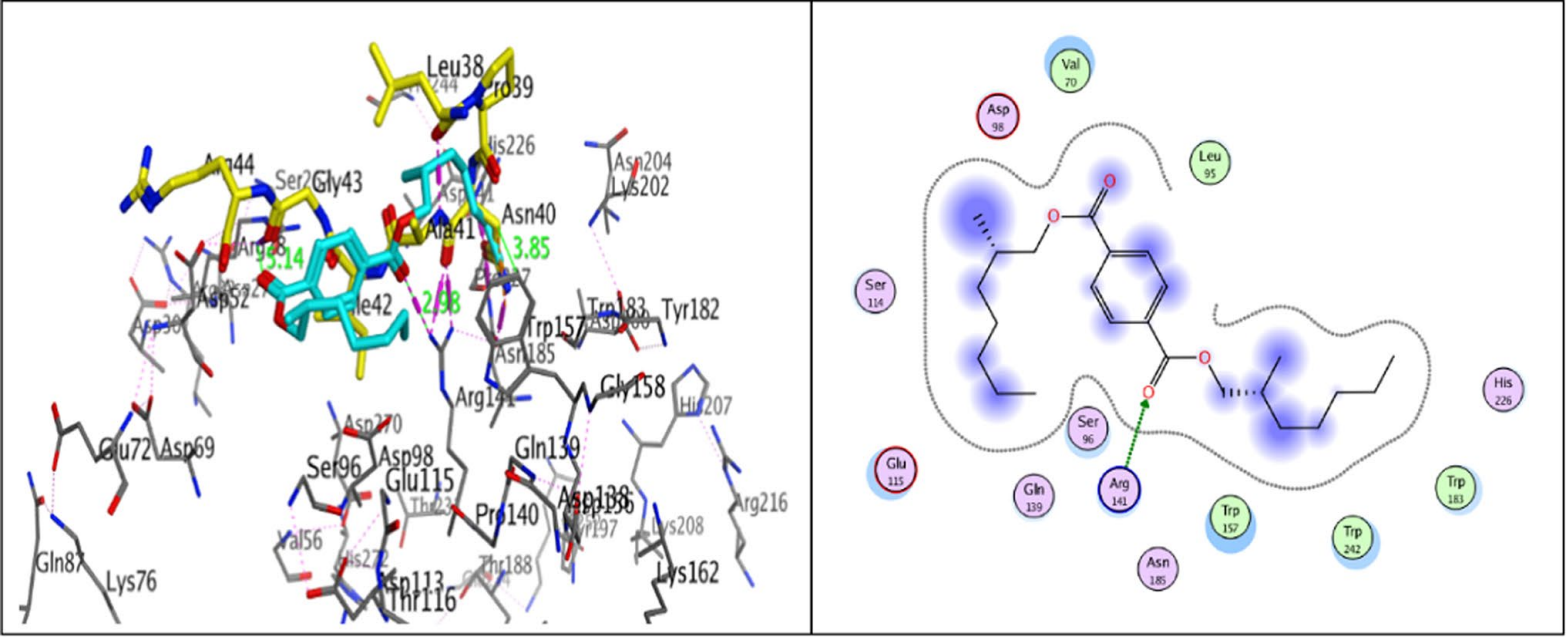
In silico studies of bis (2-methylheptyl) benzene-1,4-dicarboxylate. (**A**) The binding pose of Terephthalate (cyano) is depicted with the sclerostin peptide (yellow) on the interacting surface of LRP6 (PDB ID: 3SOV). (**B**) The binding pose of Terephthalate. 2D-structure interaction poses the essential residues of LRP6 that interact with Terephthalate.

The SOST-triggered Wnt/b-catenin pathway may serve as a promising therapeutic and adjunctive regimen against UM^74^, as evidence suggests that it can antagonize Wnt signaling in human cells by binding to the extracellular domain of LRP5/6 Wnt co-receptors and destroying Wnt-triggered Frizzled-LRP complexes^75^. Docking simulations were conducted to identify the critical interaction sites of the compound in the binding pocket of sclerostin with LRP6 to obtain insight into the binding pattern of the interaction between LRP6 and bis (2-methylheptyl) benzene-1,4-dicarboxylate. To identify the most critical residues for binding affinity, the binding site of the LRP6-sclerostin complex structure was superimposed, and the binding conformation of bis (2-methylheptyl) benzene-1,4-dicarboxylate, which was determined through docking simulation. The RMSD values were 1.40 Å, and the binding energy scores were − 5.51 kcal/mol. The 2-methylheptyl benzoate structure was positioned in the hydrophilic pocket created by Asp98, Ser114, Glu115, Gln139, and Ser96 of LRP6. The other 2-methylheptyl alkyl chain was situated in a hydrophobic cavity surrounded by Trp157, Trp183, and Trp242. This indicates that the indoles of these residues play a crucial role in developing of the peptide-protein interaction pocket. The Terephthalate core of this compound binds to the binding site of Ile within the “NXI” motif. Thus, the oxygen atom of benzene-1,4-dicarboxylate moiety form hydrogen bonds to the side chains of Arg141with distance equal 2.98 Å, which supports the prediction that this compound might bind to LRP6-E1 with high affinity (Fig. 11).

**Fig. 12:**
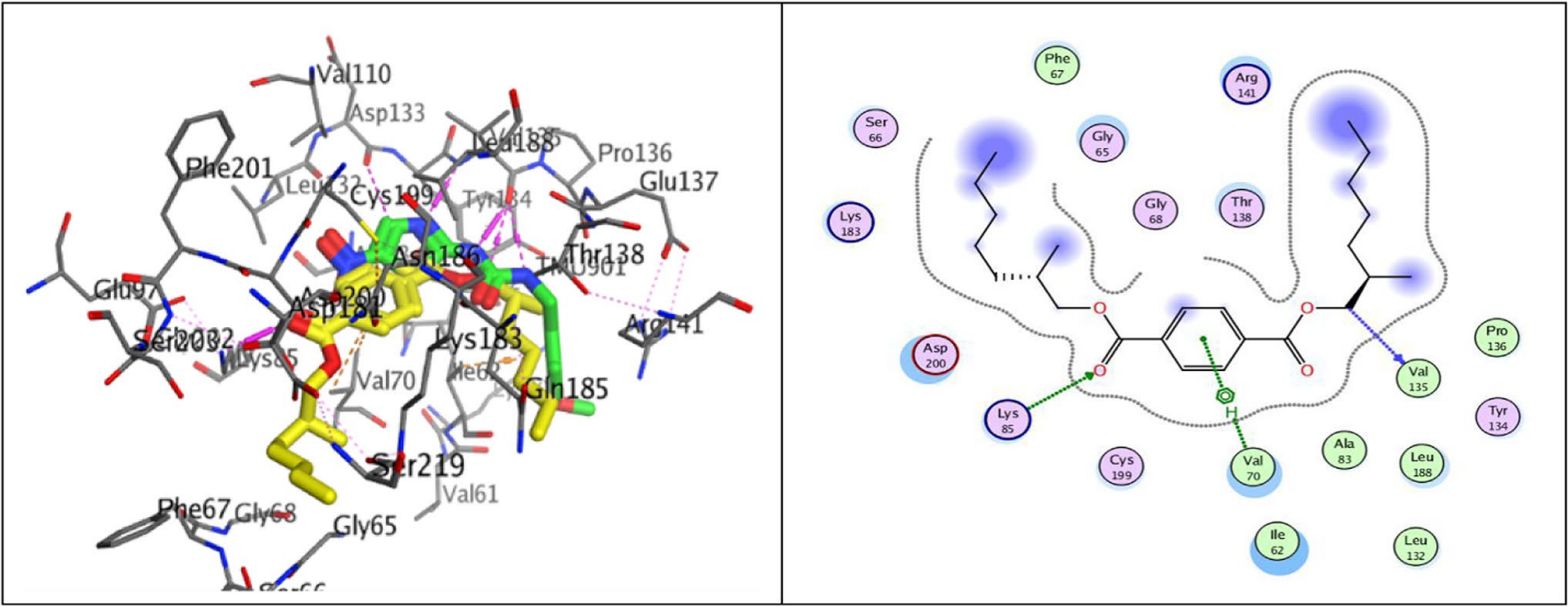
Molecular docking analysis 3D and 2D between Terephthalate with yellow color overlay the crystallized molecules with green color and GSK-3β binding pocket (PDB ID: 1Q5K). (The key residues are displayed) corresponding interactions of the docked complex (Terephthalate & GSK-3β).

The phosphorylation of β-catenin by GSK-3 is essential for signaling Wnt–β-catenin. Wnt signaling prevents the degradation of β-catenin and stabilizes it, thereby increasing the transcriptional activity of c-Myc and cyclin-D. This is achieved by inhibiting GSK-3β. GSK3β inhibitors are being investigated as potential drug candidates for various skin defects, including vitiligo^76,77^. GSK-3β comprises two primary domains: an α-helical C-terminal domain and an N-terminal β-strand. An ATP-binding site is located at the interface of these two domains and is connected by a hinge region and glycine-rich loop. Residues LYS85, GLU97, ASP113, TYR134, VAL135, THR138, ASN186, LEU188, CYS199, and ASP200 participate in the ATP binding pocket^78^. The co-crystallized structure of GSK-3β with its inhibitor AR-A014418 demonstrates that the inhibitor occupies the hinge region and the ATP pocket by forming three hydrogen bonds with residue VAL135. The docking results indicated that Terephthalate can reliably bind to the ATP pocket and superimpose the crystallized molecule with RMSD values of 1.05 Å and binding energy scores of − 7.29 kcal/mol. Further, the binding mode investigations of Terephthalate generate the primary H-bonding with Val135 at a distance of 3.38 Å and Lys 85, as well as the hydrophobic interaction with Val 70 (Fig. 12).

**Fig. 13:**
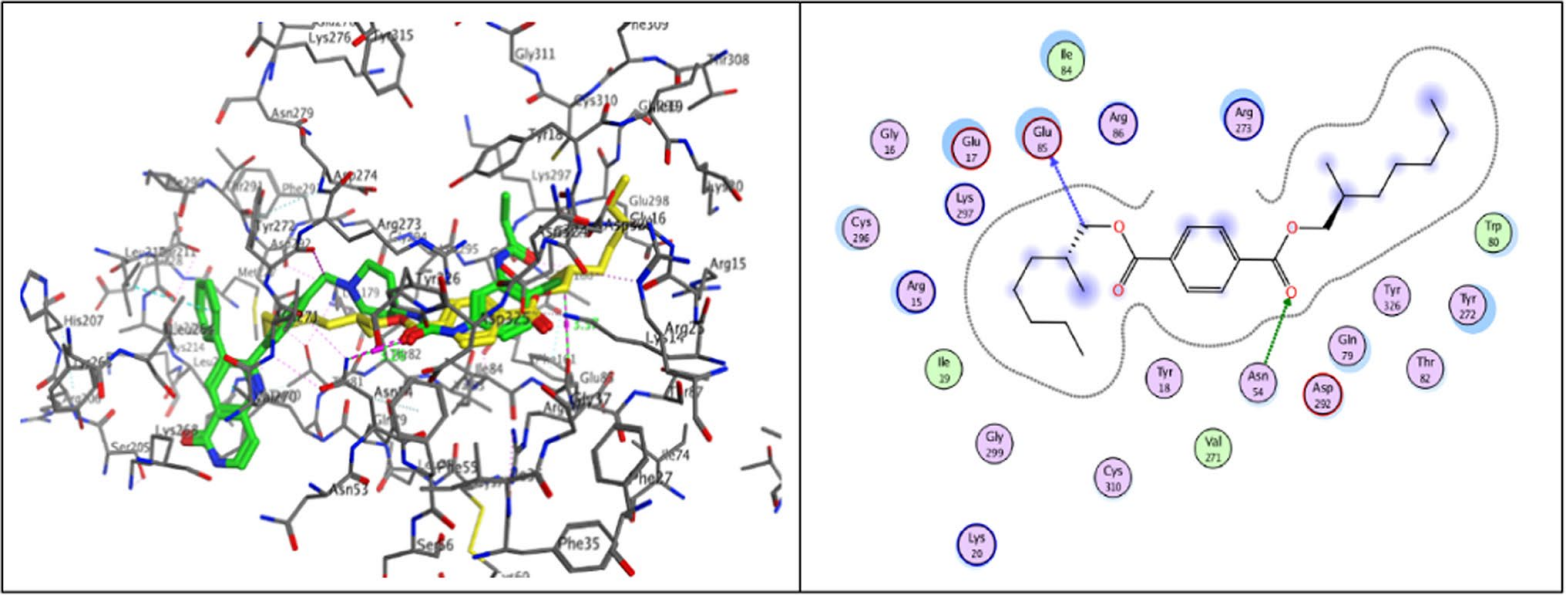
Molecular docking analysis 3D and 2D between Terephthalate with yellow color overlay the crystallized molecules with green color and ATP‐binding site of AKT-1 (PDB ID: 6HHG). (The key residues are displayed) corresponding interactions of the docked complex (Terephthalate & AKT-1).

Akt, or protein kinase B (PKB), is a target for cancer treatment. Akt stimulates cell proliferation in various complexes through phosphorylation, including glycogen synthase kinase 3 (GSK-3), TSC1/2, NF-κB, and Bcl-2 family proteins^79^. A novel dual inhibitor that has been evaluated and docked with a high potential affinity for the AKT1 target and is efficacious against normal and vemurafenib-resistant melanoma cells (PDB ID: 6HHG)^80^. Molecular docking simulations were performed to predict the potential interactions between the Terephthalate and the ATP binding pocket, which showed that the isolated natural compound occupied the binding pocket and superimposed the co-crystallized ligand with RMSD values 0.63 Å and binding energy scores of − 9.04 kcal/mol respectively. Analyzing the best pose with high binding affinity showed that alkyl substitution was close to the essential hydrophobic residue Trp80 and showed two hydrogen bonds with Asn54 and Glu85 (Fig. 13).

## Conclusion

Using diverse chromatographic methods and structure elucidation, a pure secondary metabolite was isolated from the ethyl acetate extract of a rhizosphere, *Sphingomonas sanguinis* DM. Bis (2-methylheptyl) benzene-1,4-dicarboxylate, isolated for the first time from a natural source, exhibits a noteworthy cytotoxic activity against malignant skin cells while mildly affecting normal melanocytes. In addition, the current study reveals a substantial increase in the population of early apoptotic malignant skin cells, with a moderate rise observed in late apoptotic cells compared to untreated control cells. PCR amplification and further sequencing revealed the presence of NRPS and lipopeptide *ItuD* genes in the strain, which were deposited under OR597597 and OR597598 in the NCBI GenBank database for future reference. The docking study showed that the naturally isolated compound bound at the Wnt signaling pathway protein (Fz4-CRD, LRP6, GSK3β) and Protein Kinase B/Akt binding site and superimposed the crystallized molecules with great binding affinity exceed the crystallized molecules for Fz4-CRD and GSK3β which encourage our teams to make extra mechanistic investigation to clarify the biological pathway.

## Electronic supplementary material

Below is the link to the electronic supplementary material.

Supplementary Material 1

## Data Availability

All data generated or analyzed during this study are included in this article and its supplementary files. The datasets generated during the current study are available in the NCBI GenBank database under the accession numbers OR597597 and OR597598. https://www.ncbi.nlm.nih.gov/nuccore/OR597597https://www.ncbi.nlm.nih.gov/nuccore/OR597598 16S rRNA gene sequence of bacterial strain was previously deposited in the NCBI GenBank database with the accession number PP422198. https://www.ncbi.nlm.nih.gov/nuccore/PP422198.
