# Supplementary material for: Isolation and characterization of a bioactive compound from Sphingomonas sanguinis DM with cytotoxic and molecular docking analysis
Source: Sci Rep. 2025 May 8;15:16049. doi: 10.1038/s41598-025-99178-3 (PMC12062506; doi:10.1038/s41598-025-99178-3)
Supplement: Supplementary file 1 — Supplementary Material 1 [file 41598_2025_99178_MOESM1_ESM.pdf]

Isolation and Characterization of a Bioactive Compound from *Sphingomonas sanguinis* DM with Cytotoxic and Molecular Docking Analysis

Mohamed A. Awad[1,2]

mohamed.abo-elfadl@ejust.edu.eg

Hesham S. M. Soliman[3,5*]

hesham.soliman@ejust.edu.eg

Samir F. El-Mashtoly[1,6]

samir.elmashtoly@leibniz-ipht.de

Yara E. Mansour[7]

Yara_mansour@pharm.helwan.edu.eg

Bahig El-Deeb[2]

bahig1978@gmail.com

Sherif F.Hammad[4,5]

sherif.hammad@ejust.edu.eg

[1]Biotechnology Program, Institute of Basic and Applied Science, Egypt-Japan University of Science and Technology (E-JUST), New Borg El-Arab City, 21934 Alexandria, Egypt.

[2]Botany and Microbiology Department, Faculty of Science, Sohag University, 82524 Sohag, Egypt.

[3]Department of Pharmacognosy, Faculty of Pharmacy, Helwan University, Ain-Helwan, Cairo 11795, Egypt.

[4]Department of Pharmaceutical Chemistry, Faculty of Pharmacy, Helwan University, Ain-Helwan, Cairo 11795, Egypt.

[5]PharmD Program, Egypt-Japan University of Science and Technology (E-JUST), New Borg El-Arab City, 21934 Alexandria, Egypt.

[6]Leibniz Institute of Photonic Technology, Albert-Einstein-Straße, 07745 Jena, Germany.

[7]Pharmaceutical Organic Chemistry Department, Faculty of Pharmacy, Helwan University, Ain-Helwan, Cairo 11795, Egypt.

* Corresponding author: hesham.soliman@ejust.edu.eg

Cytotoxic activities of the isolated compound

1-Evaluation of cytotoxicity against A-431 cell line

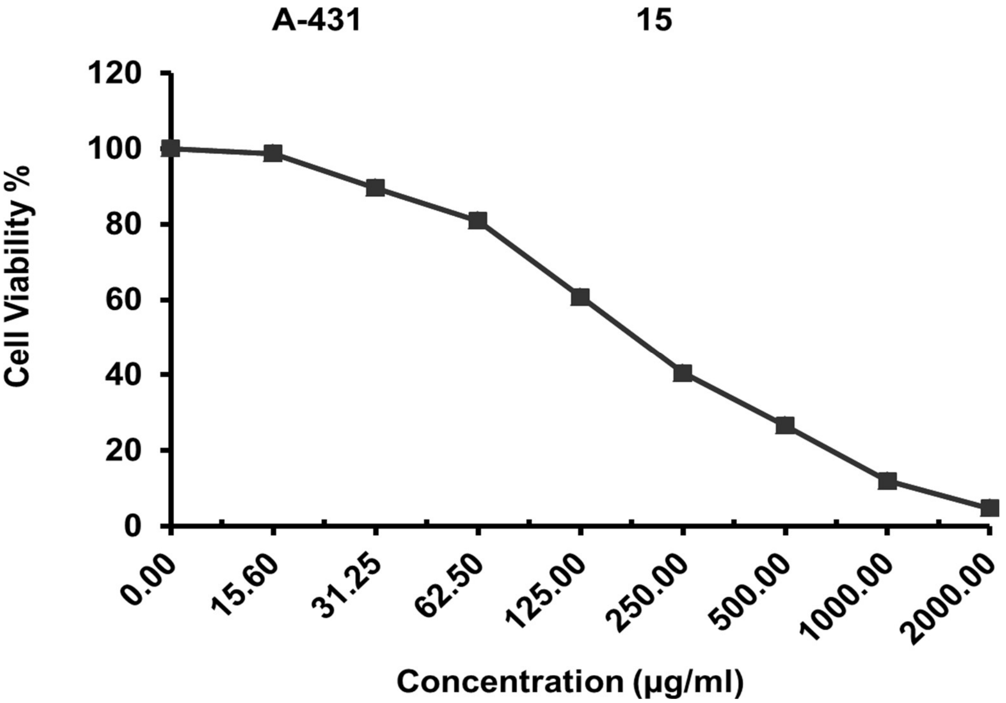

Sample conc. (µg/ml)	Viability% (3 Replicates)			Mean	Inhibitory %	S.D. (±)
	1st	2nd	3rd			
2000	4.33	5.06	4.65	4.68	95.32	0.37
1000	11.54	12.37	11.98	11.96	88.04	0.42
500	24.97	28.59	26.03	26.53	73.47	1.86
250	38.61	39.72	43.15	40.49	59.51	2.37
125	59.43	60.21	62.58	60.74	39.26	1.64
62.5	80.68	78.95	83.07	80.90	19.10	2.07
31.25	89.20	88.06	91.41	89.56	10.44	1.70
15.6	98.72	98.18	99.23	98.71	1.29	0.53
0	100	100	100	100	0	

***Comment*:** The following **IC_{50} values** (presented as three readings) were detected against **skin cell line (A-431)** using MTT assay under the experimental conditions for 48 hrs.

Sample code	IC_{50} (µg/ml) (3 Replicates)			Mean IC_{50} (µg/ml)	S.D. (±)
	1st	2nd	3rd		
15	181.62	187.29	205.93	191.61	12.72

2-Evaluation of cytotoxicity against HFB 4 cell line

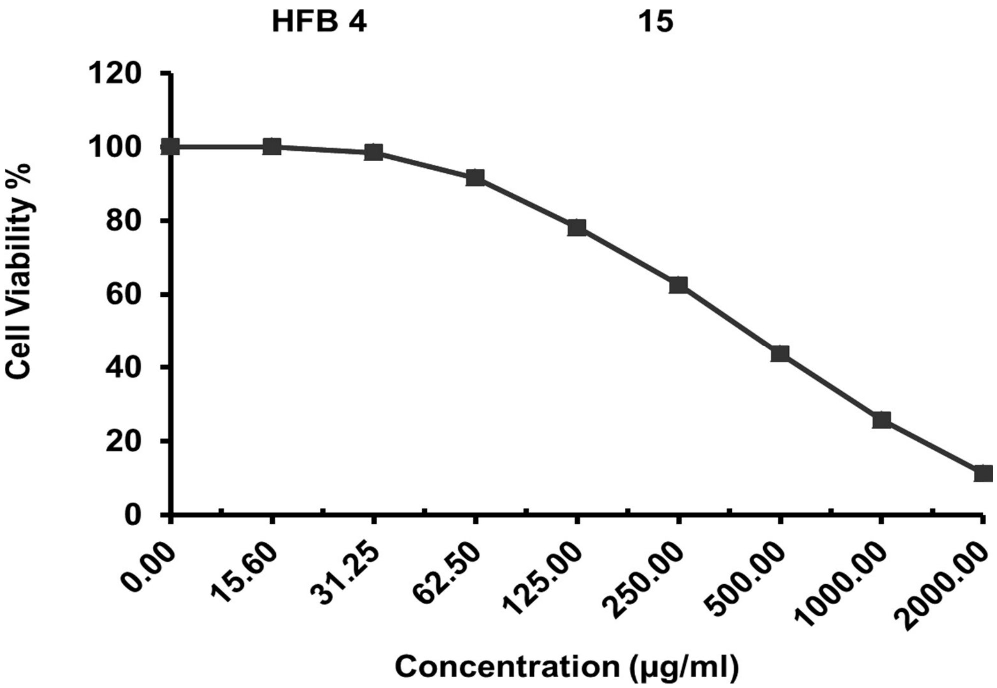

Sample conc. (µg/ml)	Viability% (3 Replicates)			Mean	Inhibitory %	S.D. (±)
	1st	2nd	3rd			
2000	12.69	11.74	9.58	11.34	88.66	1.59
1000	27.08	23.85	26.41	25.78	74.22	1.70
500	45.13	40.67	45.29	43.70	56.30	2.62
250	62.36	59.74	65.48	62.53	37.47	2.87
125	78.69	75.31	80.43	78.14	21.86	2.60
62.5	91.42	90.78	92.61	91.60	8.40	0.93
31.25	97.63	99.04	98.72	98.46	1.54	0.74
15.6	100.00	100.00	100.00	100.00	0.00	0.00
0	100	100	100	100	0	

***Comment*:** The following **IC_{50} values** (presented as three readings) were detected against **Human normal melanocytes cell line (HFB 4)** using MTT assay under the experimental conditions for 48 hrs.

Sample code	IC_{50} (µg/ml) (3 Replicates)			Mean IC_{50} (µg/ml)	S.D. (±)
	1st	2nd	3rd		
15	429.34	377.69	441.68	416.23	33.95

Apoptosis

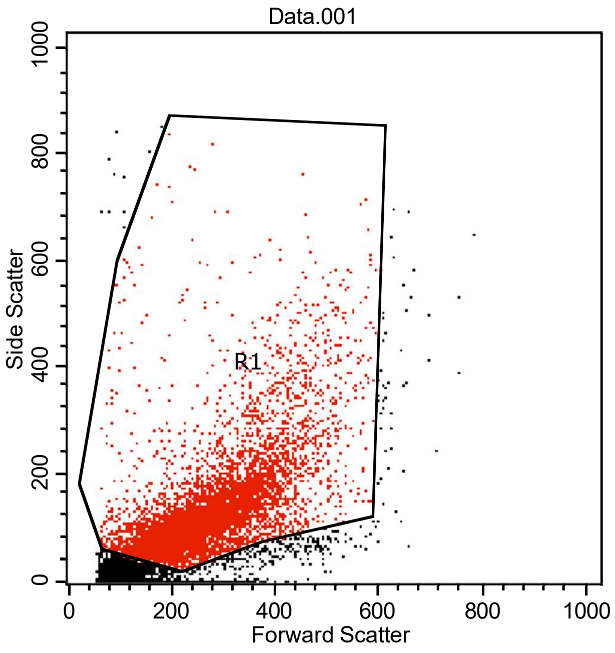

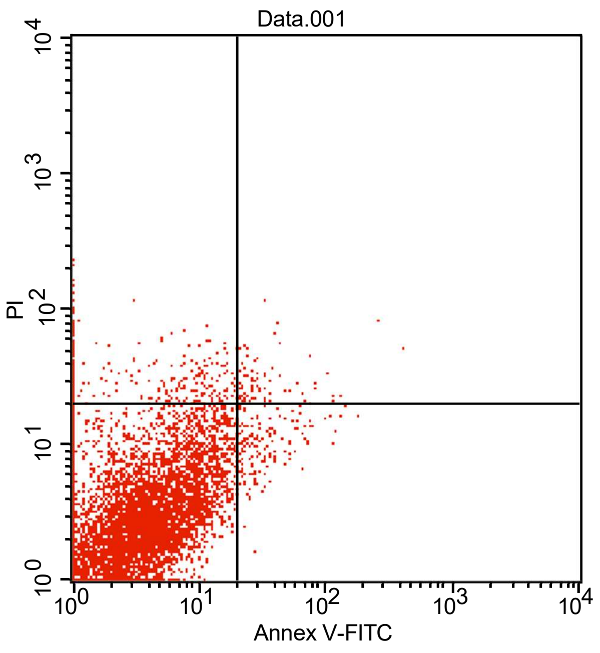

Quadrant Statistics

File: Data.001
Sample ID: Control
Tube: Untitled
Acquisition Date: 27-Feb-24
Gated Events: 7181
X Parameter: Annex V-FITC (Log)
Quad Location: 20, 20

Log Data Units: Linear Values
Patient ID: Mohamed Abo El Fadl
Panel: Untitled Acquisition Tube List
Gate: G1
Total Events: 10000
Y Parameter: PI (Log)

Quad	Events	% Gated	% Total	X Mean	X Geo Mean	Y Mean	Y Geo Mean
UL	283	3.94	2.83	5.26	2.96	38.02	32.98
UR	68	0.95	0.68	45.56	34.34	32.99	30.37
LL	6672	92.91	66.72	4.18	3.00	4.12	2.96
LR	158	2.20	1.58	36.72	32.27	10.37	9.55

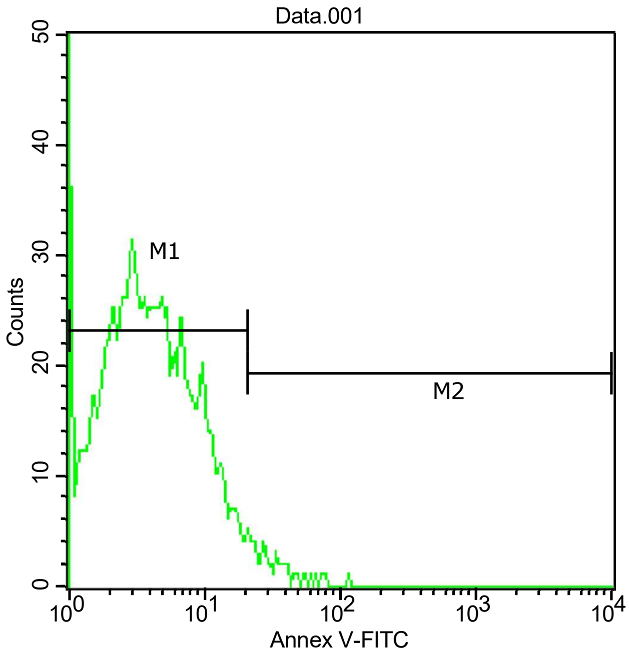

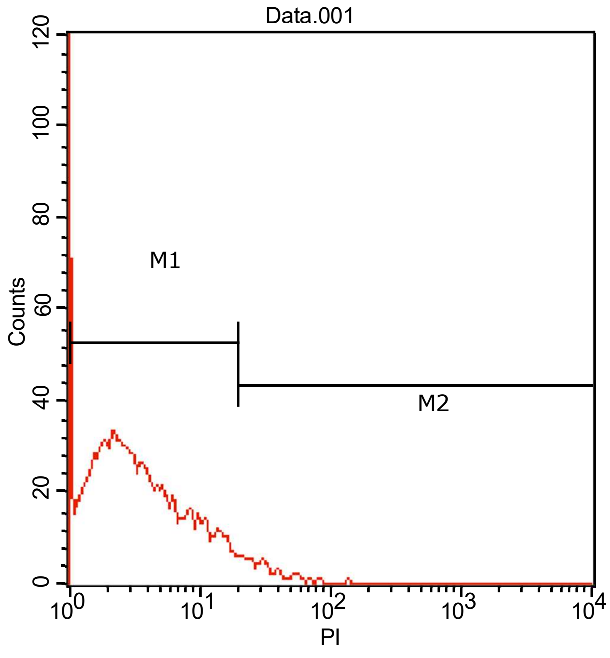

Histogram Statistics

File: Data.001
Sample ID: Control
Tube: Untitled
Acquisition Date: 27-Feb-24
Gated Events: 7181
X Parameter: Annex V-FITC (Log)

Log Data Units: Linear Values
Patient ID: Mohamed Abo El Fadl
Panel: Untitled Acquisition Tube List
Gate: G1
Total Events: 10000

Marker	Left, Right	Events	% Gated	% Total	Mean	Geo Mean	CV	Median	Peak Ch
All	1, 9910	7181	100.00	71.81	5.33	3.24	182.32	3.05	1
M1	1, 21	6976	97.15	69.76	4.28	3.02	88.69	2.94	1
M2	21, 9910	210	2.92	2.10	40.85	34.14	93.87	28.90	21

Histogram Statistics

File: Data.001
Sample ID: Control
Tube: Untitled
Acquisition Date: 27-Feb-24
Gated Events: 7181
X Parameter: PI (Log)

Log Data Units: Linear Values
Patient ID: Mohamed Abo El Fadl
Panel: Untitled Acquisition Tube List
Gate: G1
Total Events: 10000

Marker	Left, Right	Events	% Gated	% Total	Mean	Geo Mean	CV	Median	Peak Ch
All	1, 9910	7181	100.00	71.81	5.87	3.42	169.06	2.89	1
M1	1, 19	6808	94.81	68.08	4.21	3.03	90.39	2.74	1
M2	19, 9910	378	5.26	3.78	35.79	31.30	72.13	27.88	29

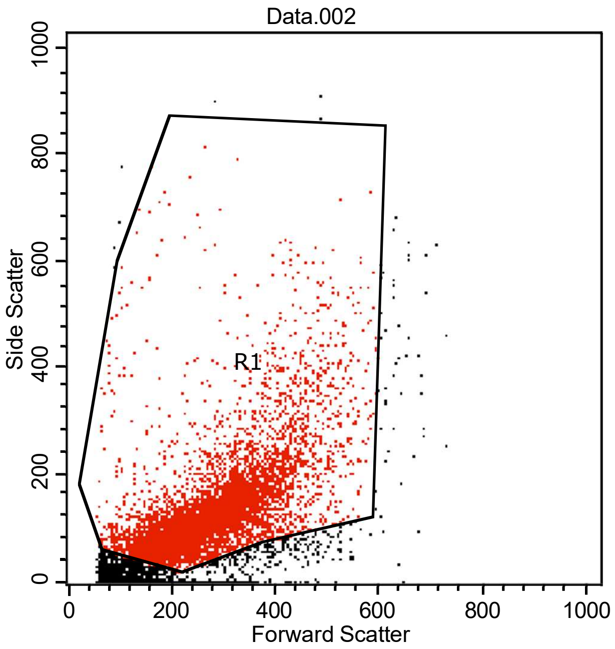

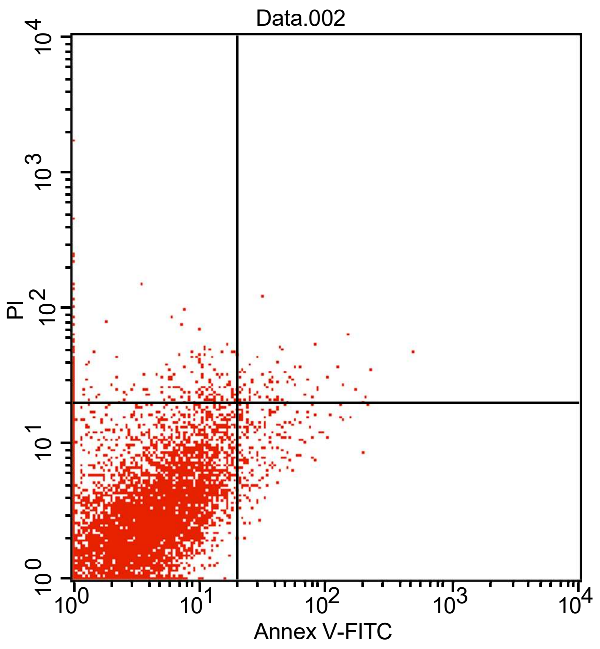

Quadrant Statistics

File: Data.002	Log Data Units: Linear Values
Sample ID: Control	Patient ID: Mohamed Abo El Fadl
Tube: Untitled	Panel: Untitled Acquisition Tube List
Acquisition Date: 27-Feb-24	Gate: G1
Gated Events: 7500	Total Events: 10000
X Parameter: Annex V-FITC (Log)	Y Parameter: PI (Log)
Quad Location: 20, 20	

Quad	Events	% Gated	% Total	X Mean	X Geo Mean	Y Mean	Y Geo Mean
UL	264	3.52	2.64	5.55	2.96	43.63	32.16
UR	69	0.92	0.69	58.02	42.22	30.00	28.26
LL	6976	93.01	69.76	4.40	3.19	3.98	2.89
LR	191	2.55	1.91	38.47	33.02	10.20	9.01

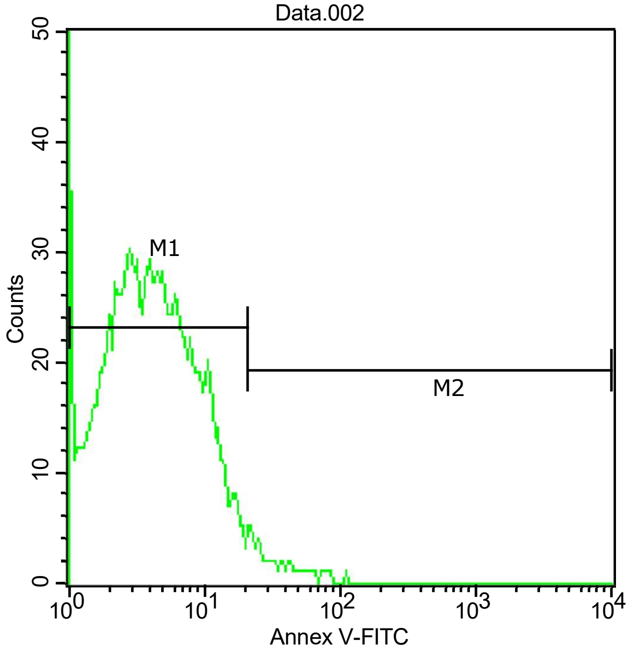

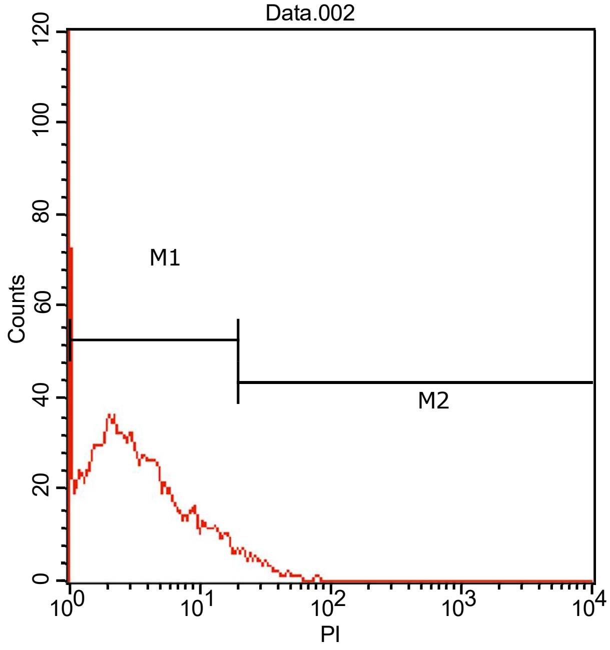

Histogram Statistics

File: Data.002
Sample ID: Control
Tube: Untitled
Acquisition Date: 27-Feb-24
Gated Events: 7500
X Parameter: Annex V-FITC (Log)

Log Data Units: Linear Values
Patient ID: Mohamed Abo El Fadl
Panel: Untitled Acquisition Tube List
Gate: G1
Total Events: 10000

Marker	Left, Right	Events	% Gated	% Total	Mean	Geo Mean	CV	Median	Peak Ch
All	1, 9910	7500	100.00	75.00	5.80	3.46	197.13	3.31	1
M1	1, 21	7258	96.77	72.58	4.48	3.20	85.57	3.16	1
M2	21, 9910	247	3.29	2.47	44.89	36.29	98.96	31.62	20

Histogram Statistics

File: Data.002
Sample ID: Control
Tube: Untitled
Acquisition Date: 27-Feb-24
Gated Events: 7500
X Parameter: PI (Log)

Log Data Units: Linear Values
Patient ID: Mohamed Abo El Fadl
Panel: Untitled Acquisition Tube List
Gate: G1
Total Events: 10000

Marker	Left, Right	Events	% Gated	% Total	Mean	Geo Mean	CV	Median	Peak Ch
All	1, 9910	7500	100.00	75.00	5.77	3.31	378.39	2.84	1
M1	1, 19	7145	95.27	71.45	4.10	2.97	90.93	2.69	1
M2	19, 9910	363	4.84	3.63	39.04	30.10	235.55	26.18	23

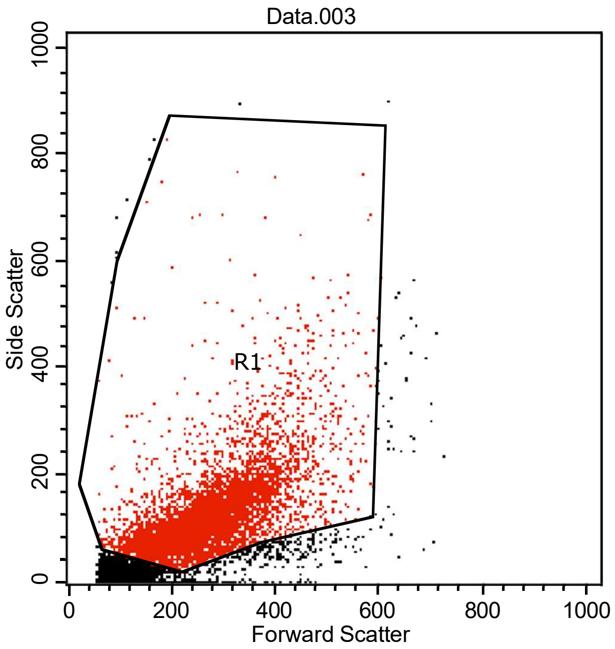

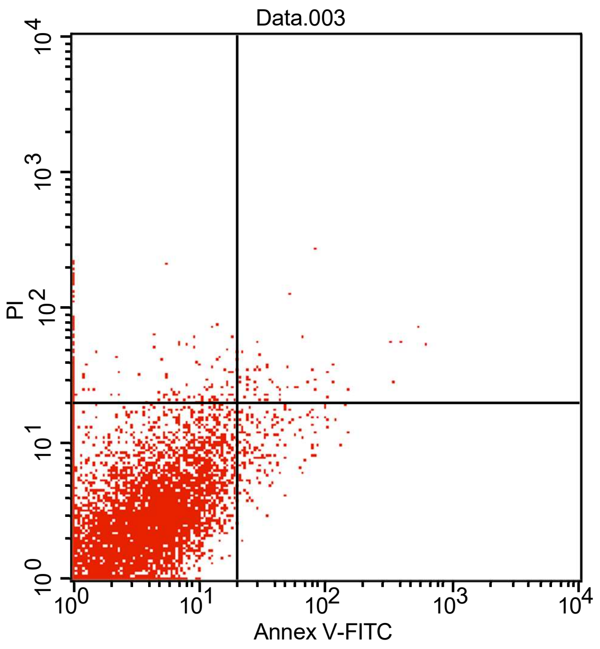

Quadrant Statistics

File: Data.003
Sample ID: Control
Tube: Untitled
Acquisition Date: 27-Feb-24
Gated Events: 7630
X Parameter: Annex V-FITC (Log)
Quad Location: 20, 20

Log Data Units: Linear Values
Patient ID: Mohamed Abo El Fadl
Panel: Untitled Acquisition Tube List
Gate: G1
Total Events: 10000
Y Parameter: PI (Log)

Quad	Events	% Gated	% Total	X Mean	X Geo Mean	Y Mean	Y Geo Mean
UL	201	2.63	2.01	4.69	2.38	40.38	33.42
UR	54	0.71	0.54	82.71	49.87	37.50	32.03
LL	7206	94.44	72.06	4.22	3.07	3.73	2.76
LR	169	2.21	1.69	38.88	34.08	10.46	9.55

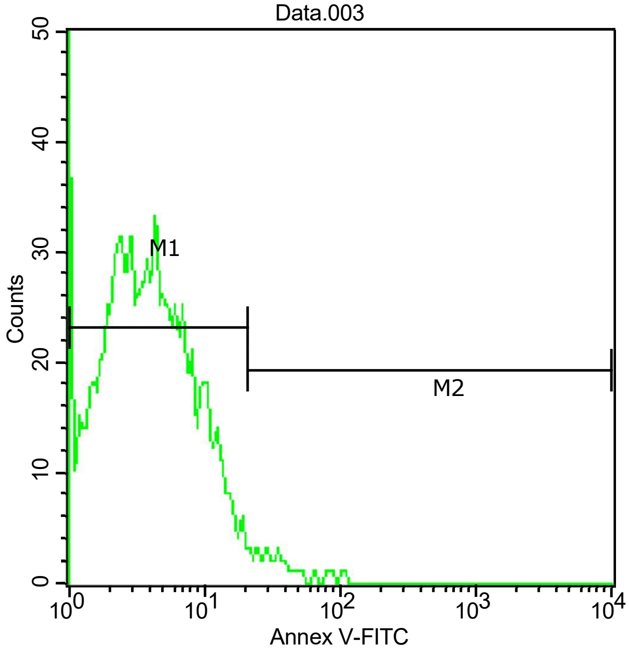

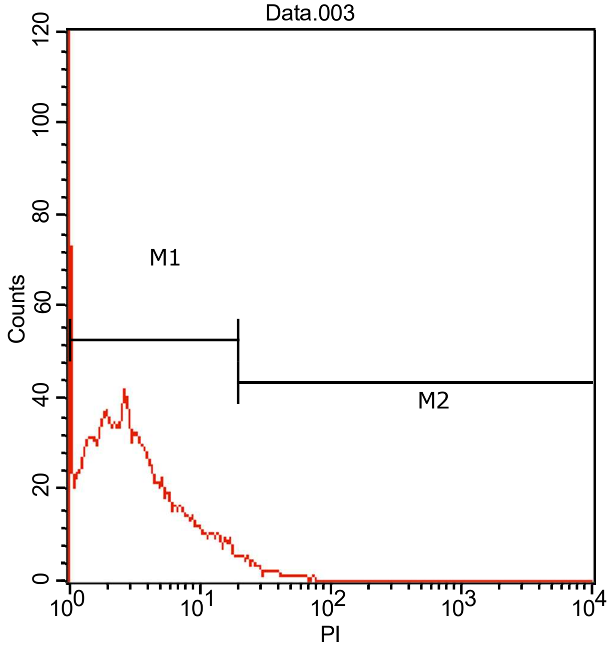

Histogram Statistics

File: Data.003
Sample ID: Control
Tube: Untitled
Acquisition Date: 27-Feb-24
Gated Events: 7630
X Parameter: Annex V-FITC (Log)

Log Data Units: Linear Values
Patient ID: Mohamed Abo El Fadl
Panel: Untitled Acquisition Tube List
Gate: G1
Total Events: 10000

Marker	Left, Right	Events	% Gated	% Total	Mean	Geo Mean	CV	Median	Peak Ch
All	1, 9910	7630	100.00	76.30	5.56	3.28	252.86	3.11	1
M1	1, 21	7422	97.27	74.22	4.27	3.06	86.42	3.00	1
M2	21, 9910	211	2.77	2.11	51.17	38.71	131.75	32.49	23

Histogram Statistics

File: Data.003
Sample ID: Control
Tube: Untitled
Acquisition Date: 27-Feb-24
Gated Events: 7630
X Parameter: PI (Log)

Log Data Units: Linear Values
Patient ID: Mohamed Abo El Fadl
Panel: Untitled Acquisition Tube List
Gate: G1
Total Events: 10000

Marker	Left, Right	Events	% Gated	% Total	Mean	Geo Mean	CV	Median	Peak Ch
All	1, 9910	7630	100.00	76.30	5.08	3.08	191.46	2.67	1
M1	1, 19	7361	96.47	73.61	3.85	2.83	91.02	2.57	1
M2	19, 9910	272	3.56	2.72	38.51	32.04	88.91	27.26	25

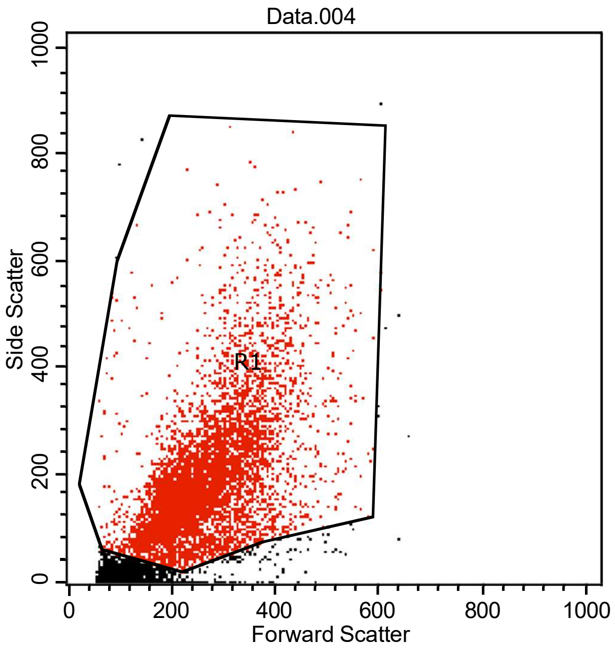

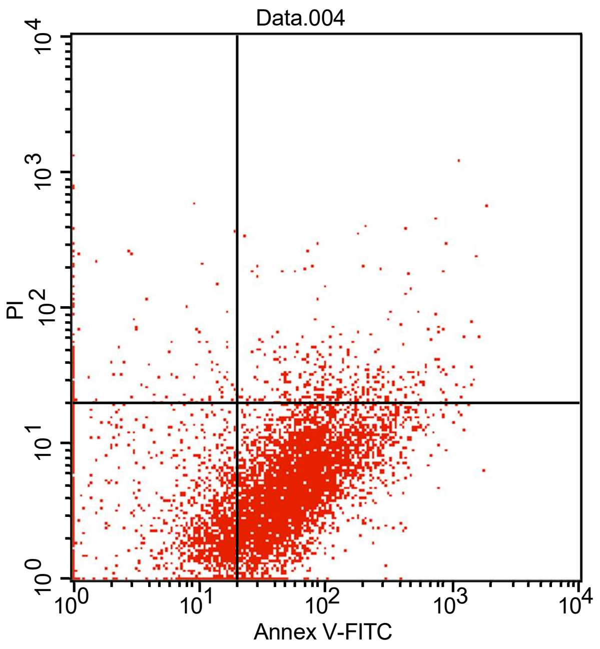

Quadrant Statistics

File: Data.004
Sample ID: Treated
Tube: Untitled
Acquisition Date: 27-Feb-24
Gated Events: 7498
X Parameter: Annex V-FITC (Log)
Quad Location: 20, 20

Log Data Units: Linear Values
Patient ID: Mohamed Abo El Fadl
Panel: Untitled Acquisition Tube List
Gate: G1
Total Events: 10000
Y Parameter: PI (Log)

Quad	Events	% Gated	% Total	X Mean	X Geo Mean	Y Mean	Y Geo Mean
UL	153	2.04	1.53	4.25	2.22	82.79	46.24
UR	350	4.67	3.50	238.17	142.02	50.61	35.35
LL	1579	21.06	15.79	10.20	6.90	4.01	2.77
LR	5416	72.23	54.16	79.74	58.73	5.87	4.70

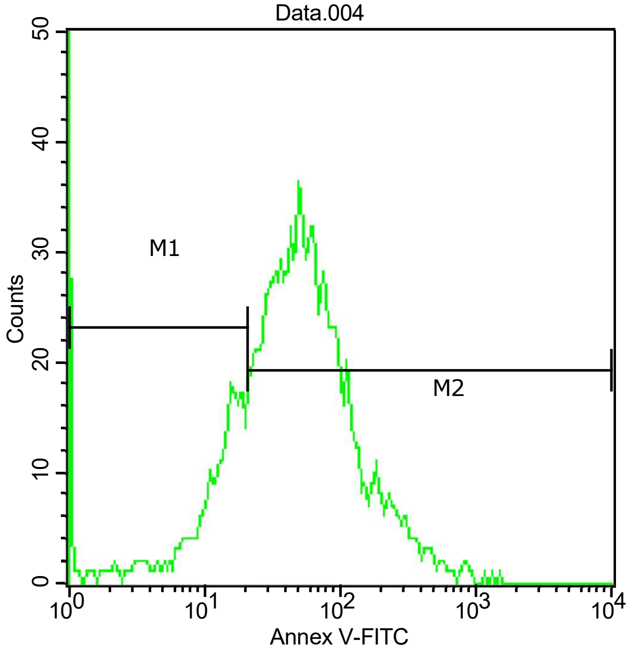

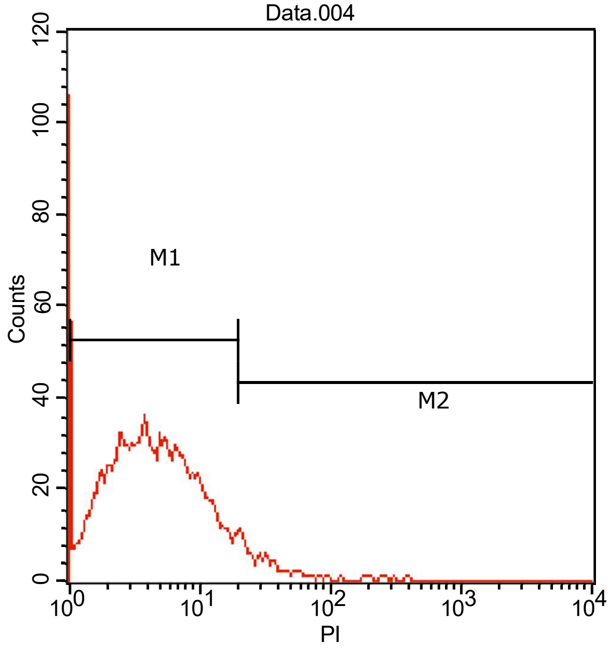

Histogram Statistics

File: Data.004
Sample ID: Treated
Tube: Untitled
Acquisition Date: 27-Feb-24
Gated Events: 7498
X Parameter: Annex V-FITC (Log)

Log Data Units: Linear Values
Patient ID: Mohamed Abo El Fadl
Panel: Untitled Acquisition Tube List
Gate: G1
Total Events: 10000

Marker	Left, Right	Events	% Gated	% Total	Mean	Geo Mean	CV	Median	Peak Ch
All	1, 9910	7498	100.00	74.98	70.95	36.46	155.12	42.55	1
M1	1, 21	1805	24.07	18.05	10.10	6.54	65.44	11.14	1
M2	21, 9910	5709	76.14	57.09	90.05	62.66	133.11	55.73	47

Histogram Statistics

File: Data.004
Sample ID: Treated
Tube: Untitled
Acquisition Date: 27-Feb-24
Gated Events: 7498
X Parameter: PI (Log)

Log Data Units: Linear Values
Patient ID: Mohamed Abo El Fadl
Panel: Untitled Acquisition Tube List
Gate: G1
Total Events: 10000

Marker	Left, Right	Events	% Gated	% Total	Mean	Geo Mean	CV	Median	Peak Ch
All	1, 9910	7498	100.00	74.98	9.13	4.84	349.29	4.41	1
M1	1, 19	6950	92.69	69.50	5.36	4.13	74.05	4.03	1
M2	19, 9910	559	7.46	5.59	56.30	35.85	186.94	28.13	19

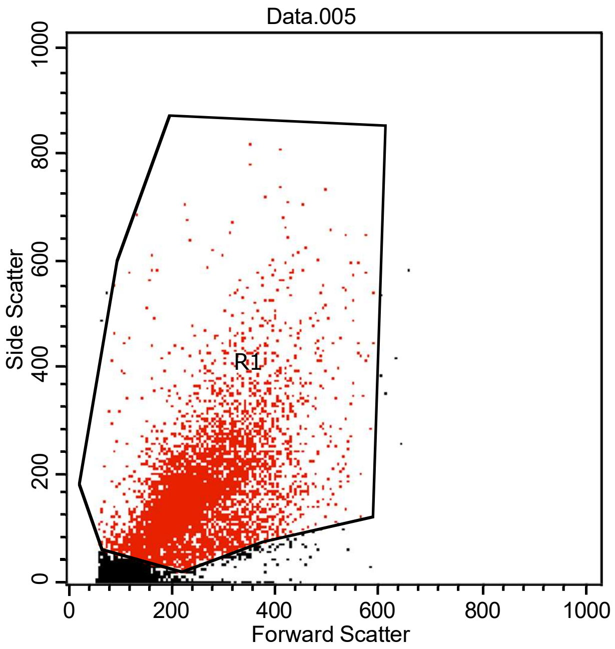

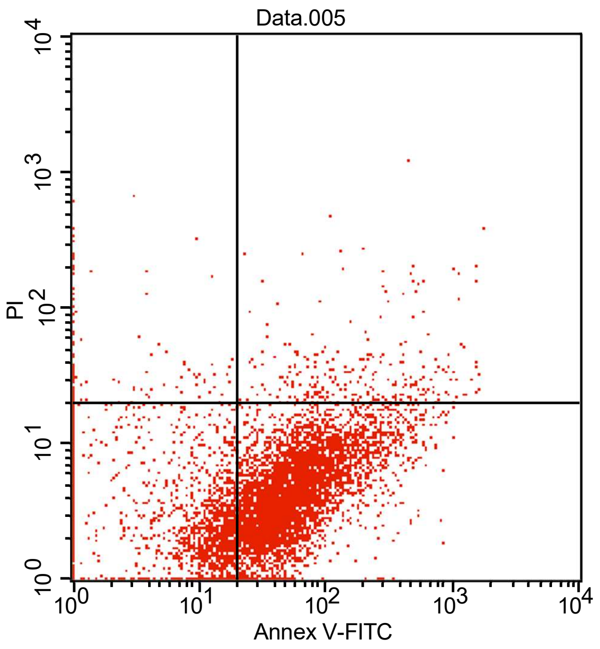

Quadrant Statistics

File: Data.005
Sample ID: Treated
Tube: Untitled
Acquisition Date: 27-Feb-24
Gated Events: 7093
X Parameter: Annex V-FITC (Log)
Quad Location: 20, 20

Log Data Units: Linear Values
Patient ID: Mohamed Abo El Fadl
Panel: Untitled Acquisition Tube List
Gate: G1
Total Events: 10000
Y Parameter: PI (Log)

Quad	Events	% Gated	% Total	X Mean	X Geo Mean	Y Mean	Y Geo Mean
UL	120	1.69	1.20	3.43	1.94	76.05	46.31
UR	250	3.52	2.50	333.74	192.18	52.35	36.35
LL	1693	23.87	16.93	9.83	6.52	3.88	2.68
LR	5030	70.91	50.30	77.82	56.76	5.20	4.19

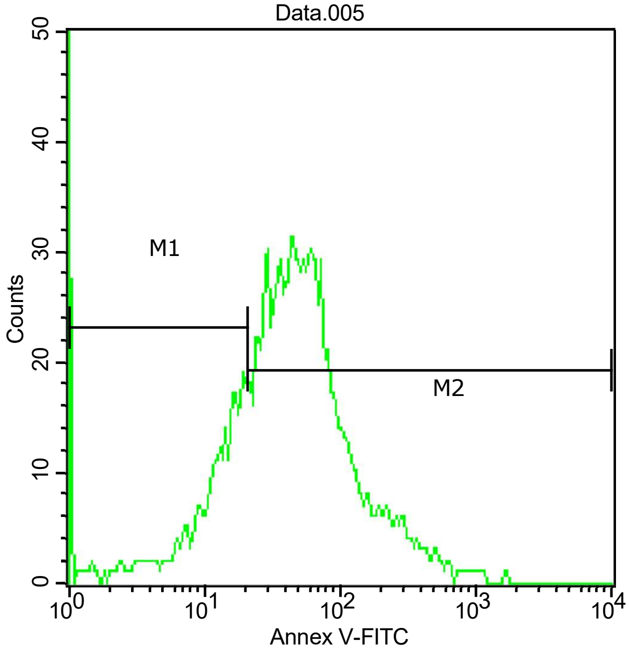

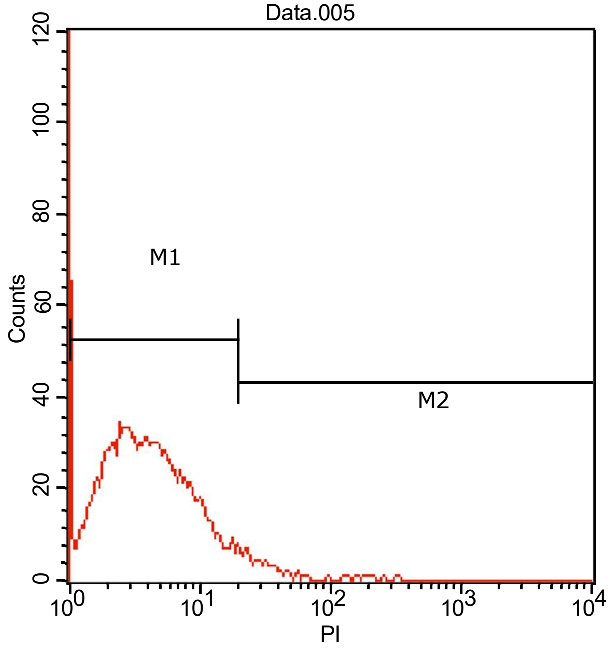

Histogram Statistics

File: Data.005
Sample ID: Treated
Tube: Untitled
Acquisition Date: 27-Feb-24
Gated Events: 7093
X Parameter: Annex V-FITC (Log)

Log Data Units: Linear Values
Patient ID: Mohamed Abo El Fadl
Panel: Untitled Acquisition Tube List
Gate: G1
Total Events: 10000

Marker	Left, Right	Events	% Gated	% Total	Mean	Geo Mean	CV	Median	Peak Ch
All	1, 9910	7093	100.00	70.93	69.35	33.39	167.83	39.24	1
M1	1, 21	1897	26.74	18.97	9.89	6.35	67.45	10.84	1
M2	21, 9910	5212	73.48	52.12	90.85	61.00	142.17	52.80	56

Histogram Statistics

File: Data.005
Sample ID: Treated
Tube: Untitled
Acquisition Date: 27-Feb-24
Gated Events: 7093
X Parameter: PI (Log)

Log Data Units: Linear Values
Patient ID: Mohamed Abo El Fadl
Panel: Untitled Acquisition Tube List
Gate: G1
Total Events: 10000

Marker	Left, Right	Events	% Gated	% Total	Mean	Geo Mean	CV	Median	Peak Ch
All	1, 9910	7093	100.00	70.93	7.75	4.23	326.09	3.85	1
M1	1, 19	6699	94.45	66.99	4.82	3.72	76.47	3.62	1
M2	19, 9910	402	5.67	4.02	56.81	37.18	162.42	28.64	22

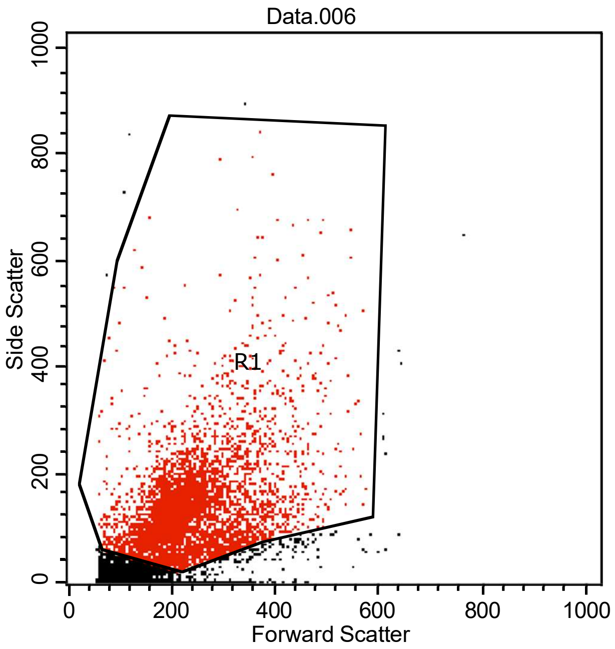

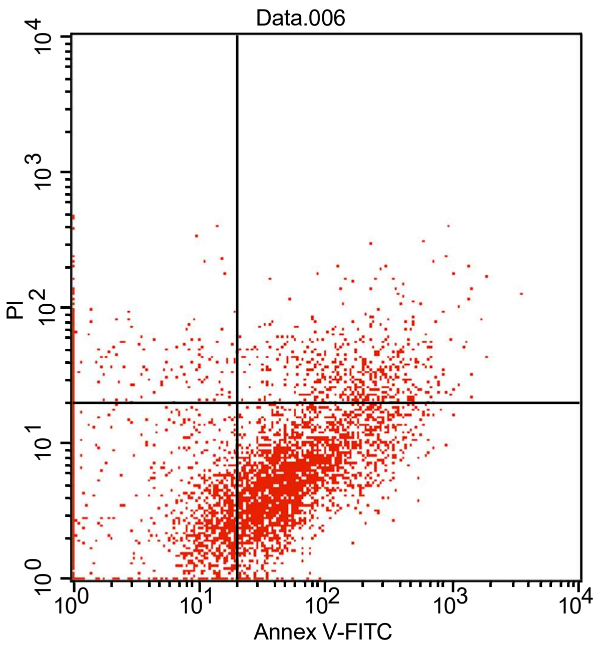

Quadrant Statistics

File: Data.006
Sample ID: Treated
Tube: Untitled
Acquisition Date: 27-Feb-24
Gated Events: 5096
X Parameter: Annex V-FITC (Log)
Quad Location: 20, 20

Log Data Units: Linear Values
Patient ID: Mohamed Abo El Fadl
Panel: Untitled Acquisition Tube List
Gate: G1
Total Events: 10000
Y Parameter: PI (Log)

Quad	Events	% Gated	% Total	X Mean	X Geo Mean	Y Mean	Y Geo Mean
UL	300	5.89	3.00	3.20	1.78	53.17	43.20
UR	517	10.15	5.17	261.45	173.80	44.70	37.52
LL	1393	27.34	13.93	8.88	5.20	3.97	2.87
LR	2886	56.63	28.86	81.21	57.32	6.50	5.32

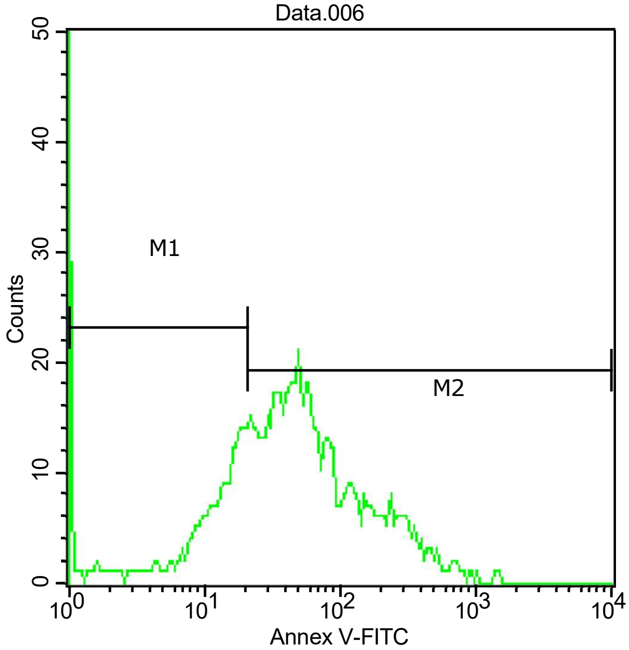

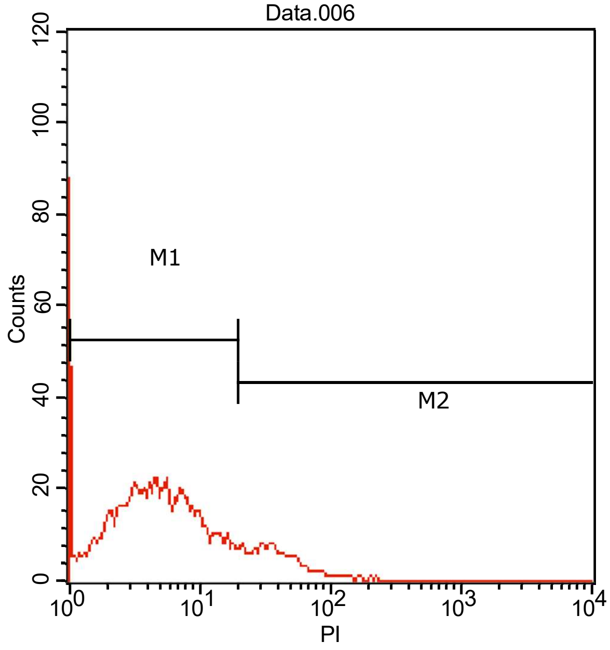

Histogram Statistics

File: Data.006
Sample ID: Treated
Tube: Untitled
Acquisition Date: 27-Feb-24
Gated Events: 5096
X Parameter: Annex V-FITC (Log)

Log Data Units: Linear Values
Patient ID: Mohamed Abo El Fadl
Panel: Untitled Acquisition Tube List
Gate: G1
Total Events: 10000

Marker	Left, Right	Events	% Gated	% Total	Mean	Geo Mean	CV	Median	Peak Ch
All	1, 9910	5096	100.00	50.96	75.13	27.14	180.70	34.60	1
M1	1, 21	1757	34.48	17.57	8.32	4.55	83.78	8.06	1
M2	21, 9910	3349	65.72	33.49	110.02	69.19	142.19	56.23	47

Histogram Statistics

File: Data.006
Sample ID: Treated
Tube: Untitled
Acquisition Date: 27-Feb-24
Gated Events: 5096
X Parameter: PI (Log)

Log Data Units: Linear Values
Patient ID: Mohamed Abo El Fadl
Panel: Untitled Acquisition Tube List
Gate: G1
Total Events: 10000

Marker	Left, Right	Events	% Gated	% Total	Mean	Geo Mean	CV	Median	Peak Ch
All	1, 9910	5096	100.00	50.96	12.44	6.20	193.69	5.33	1
M1	1, 19	4250	83.40	42.50	5.59	4.31	73.06	4.37	1
M2	19, 9910	850	16.68	8.50	46.71	38.44	95.43	34.91	20

Characterization and Identification of the isolated Compound via Structure Elucidation

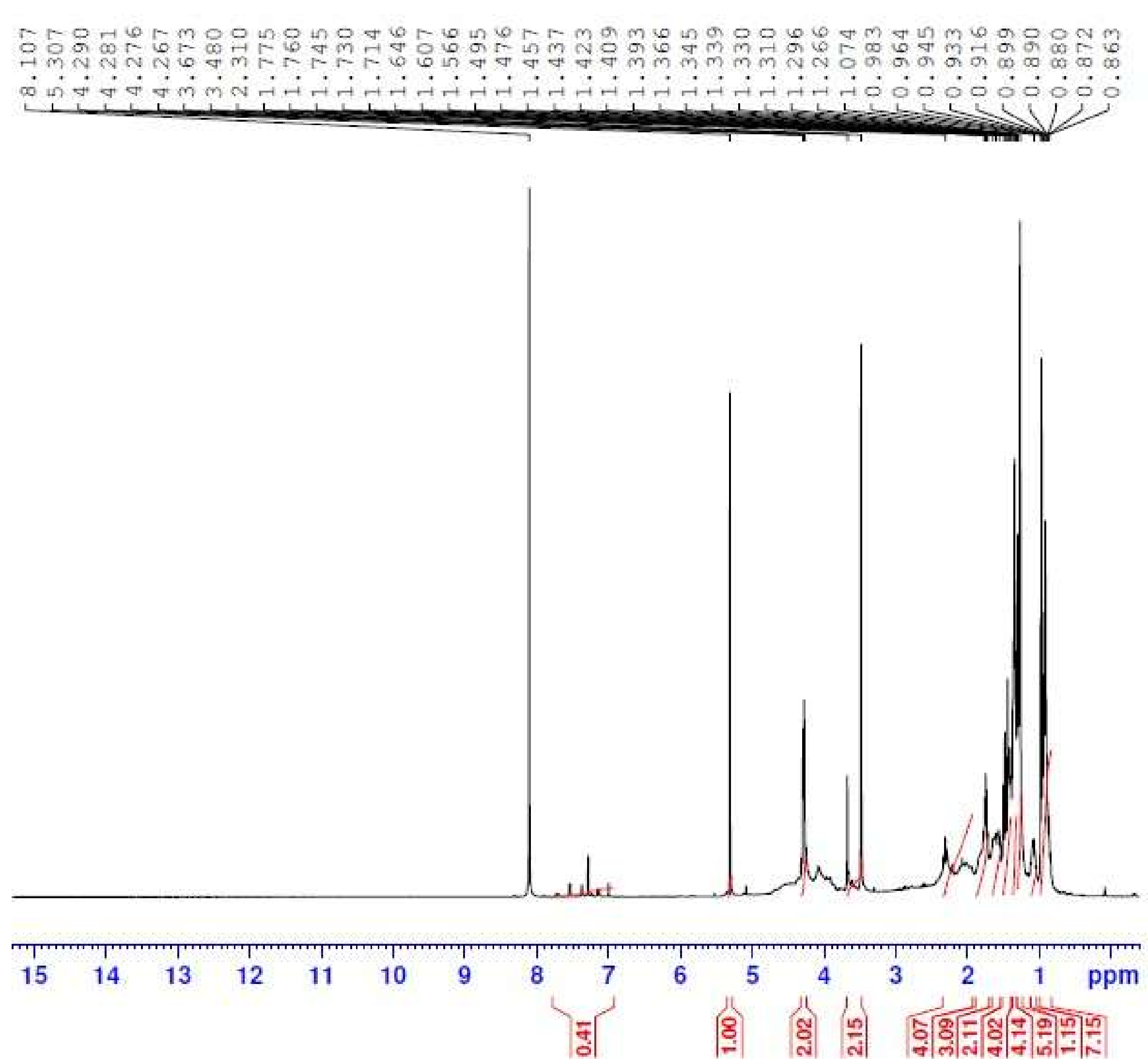

Fig. S1 ^{1}H NMR (400 MHz, CDCl3) spectrum of the isolated compound

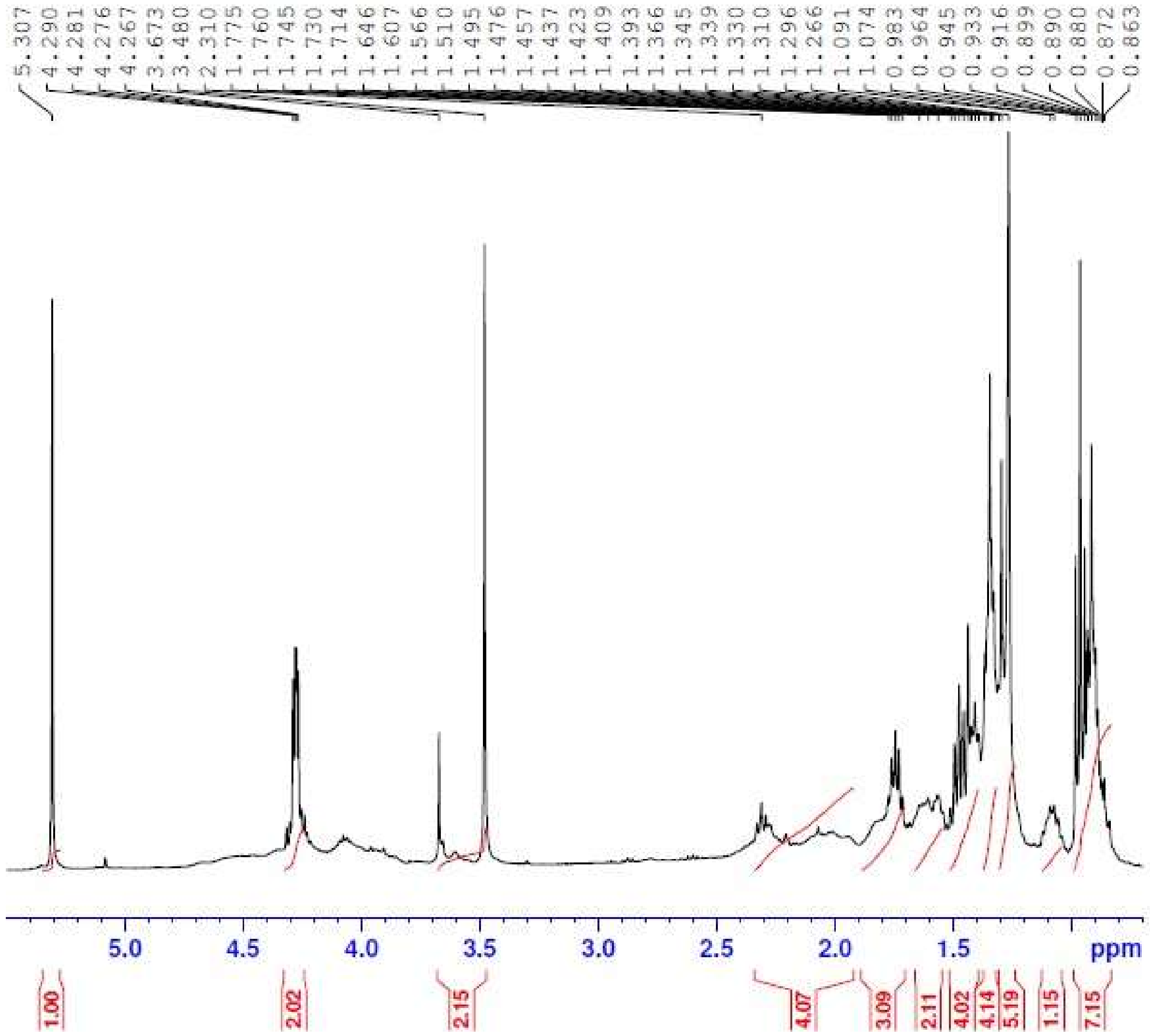

Fig. S2 Magnification of ^{1}H NMR (400 MHz, CDCl3) spectrum of the isolated compound (δ ppm 1.5-5.0)

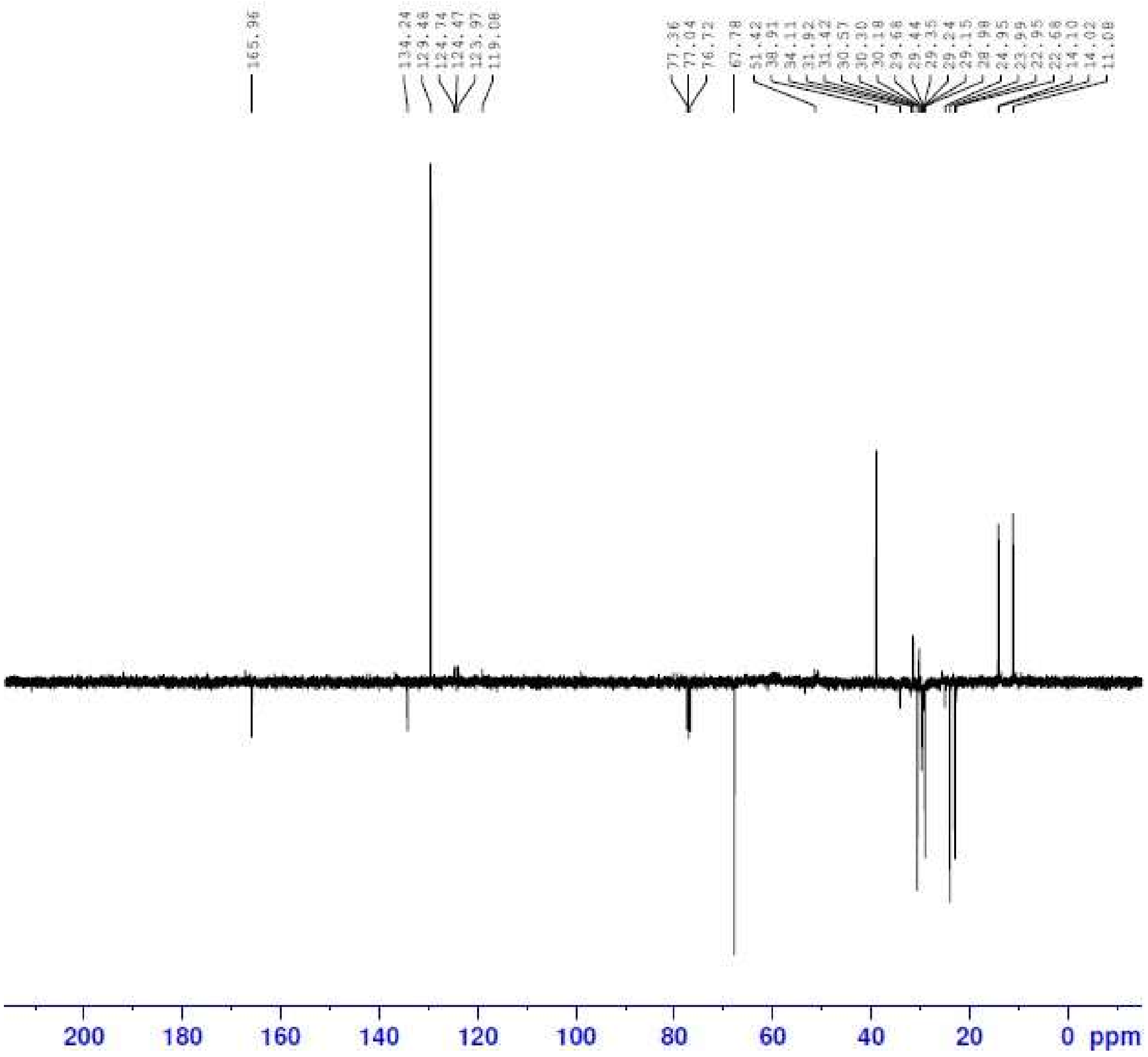

Fig. S3 APT ^{13}C NMR (100.63 MHz, CDCl3), spectrum of the isolated compound

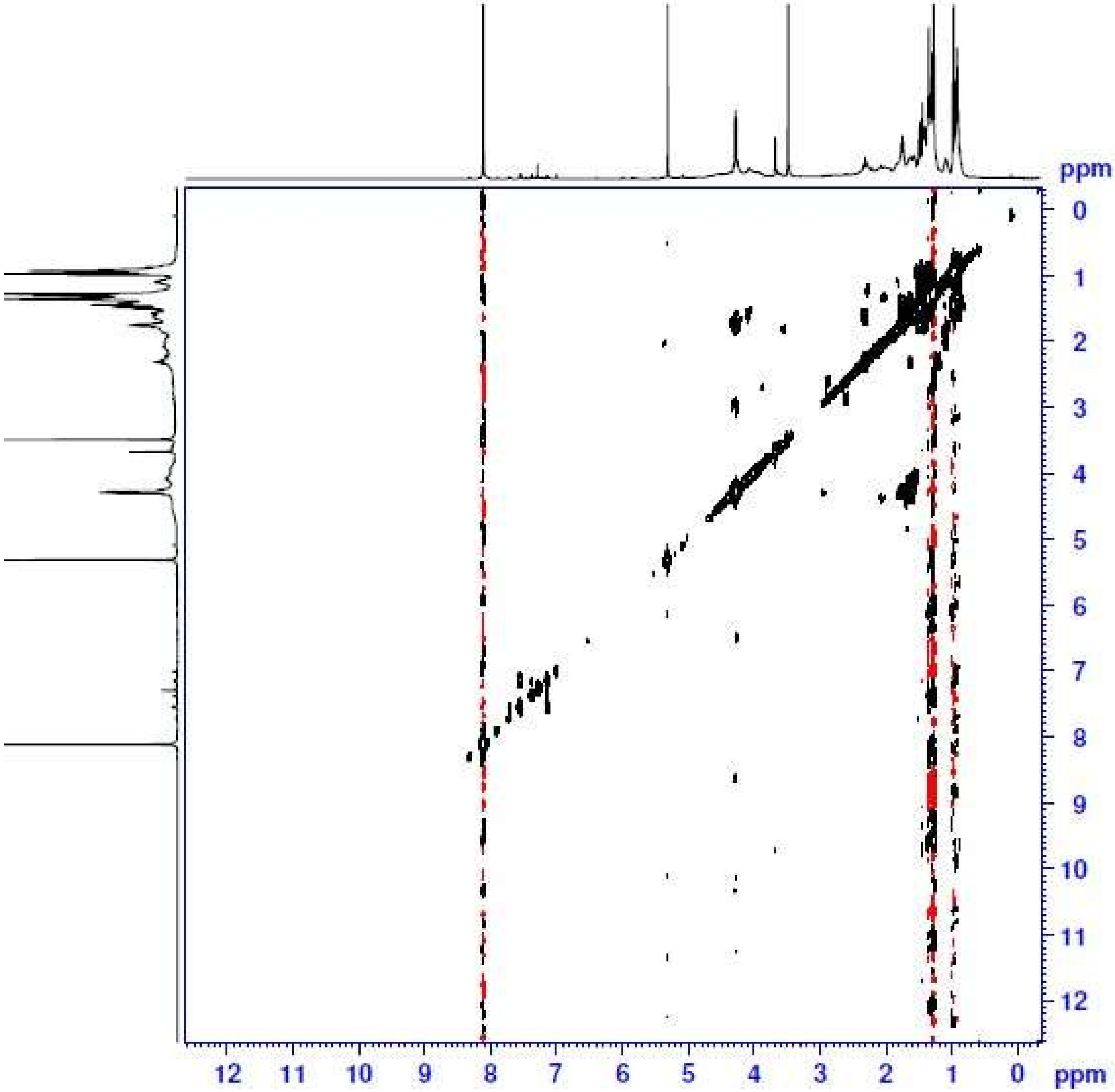

Fig. S4 H-H COSY-1 spectrum of the isolated compound

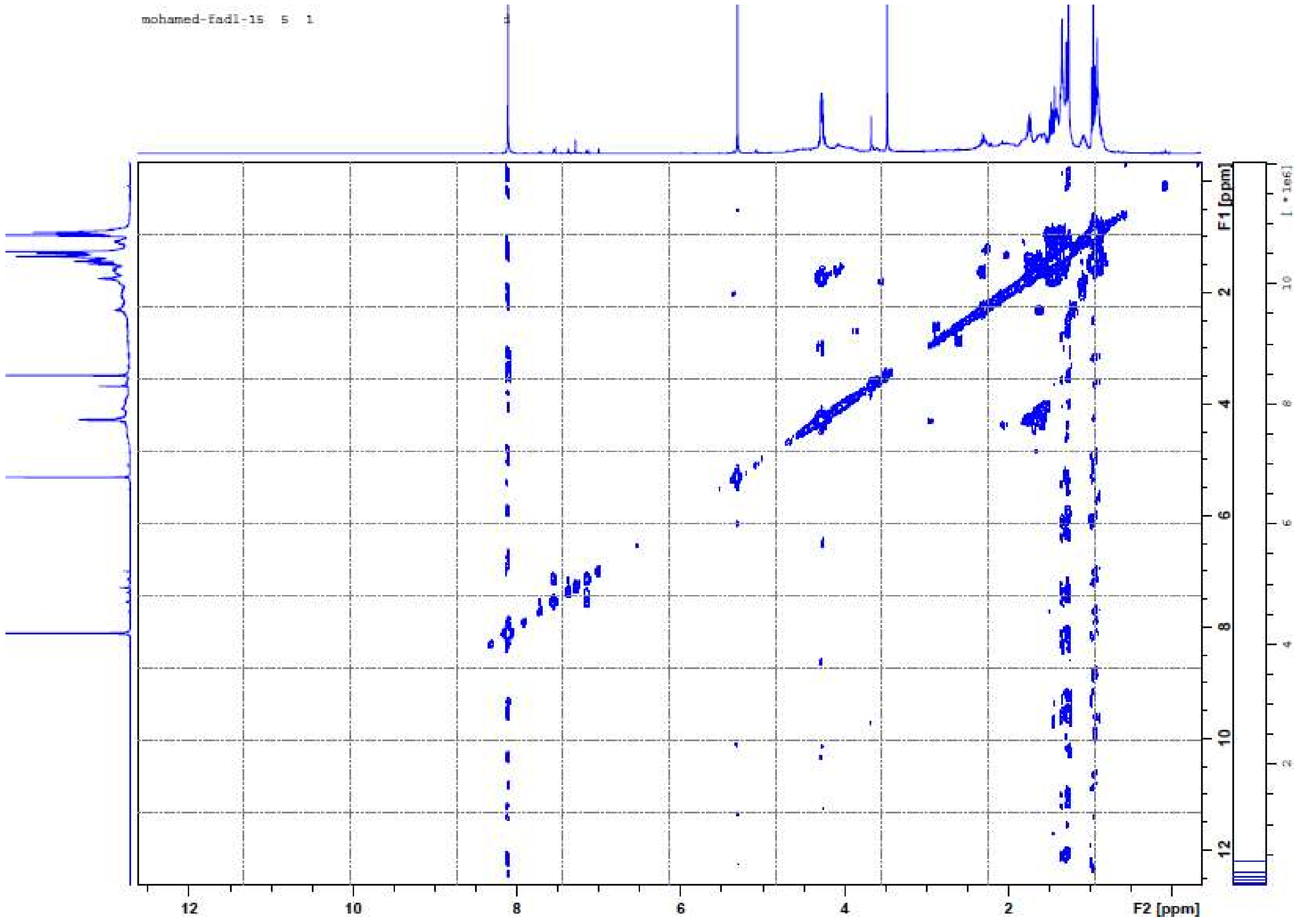

Fig. S5 H-H COSY-2 spectrum of the isolated compound

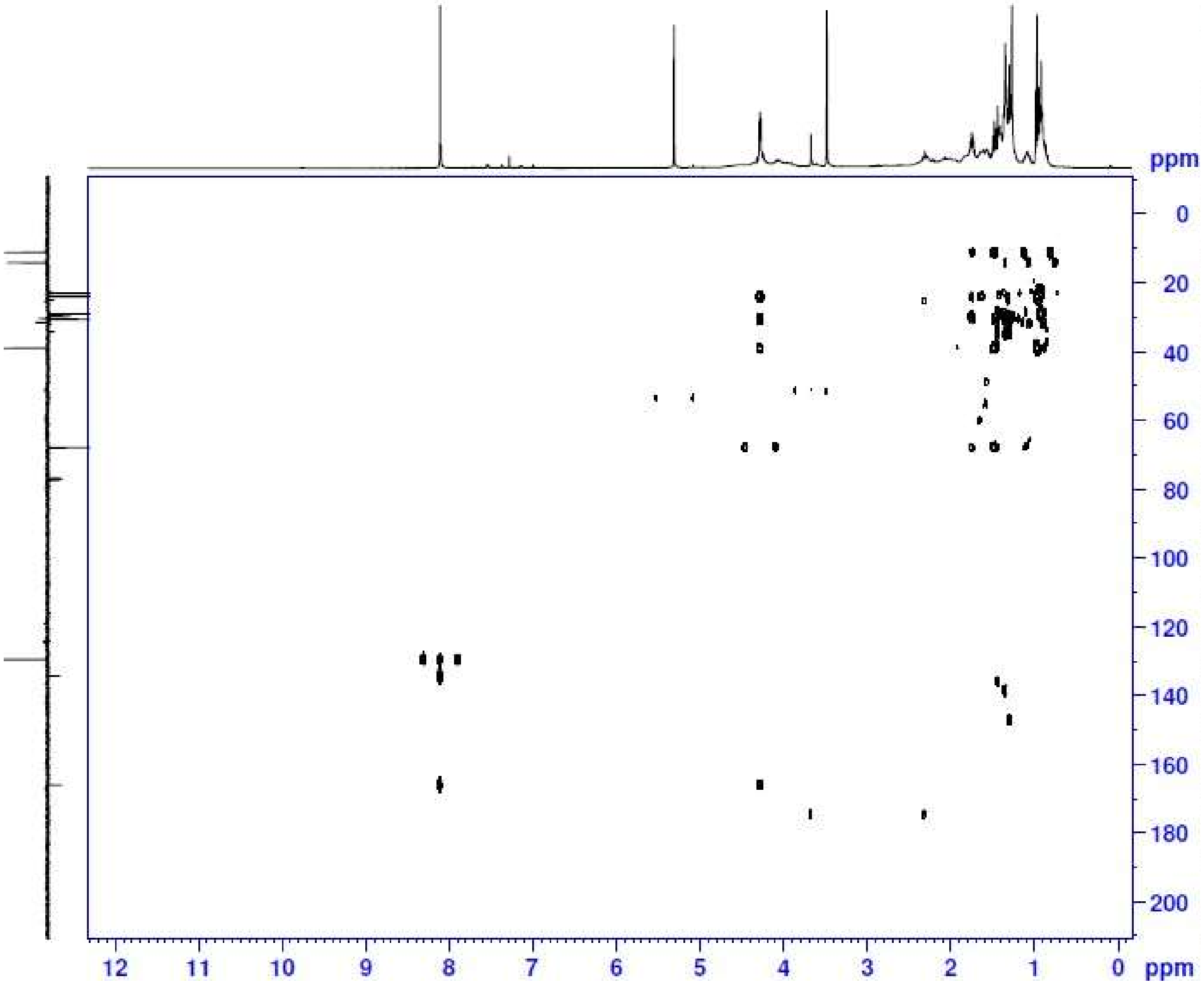

Fig. S6 HMBC-1 spectrum of the isolated compound

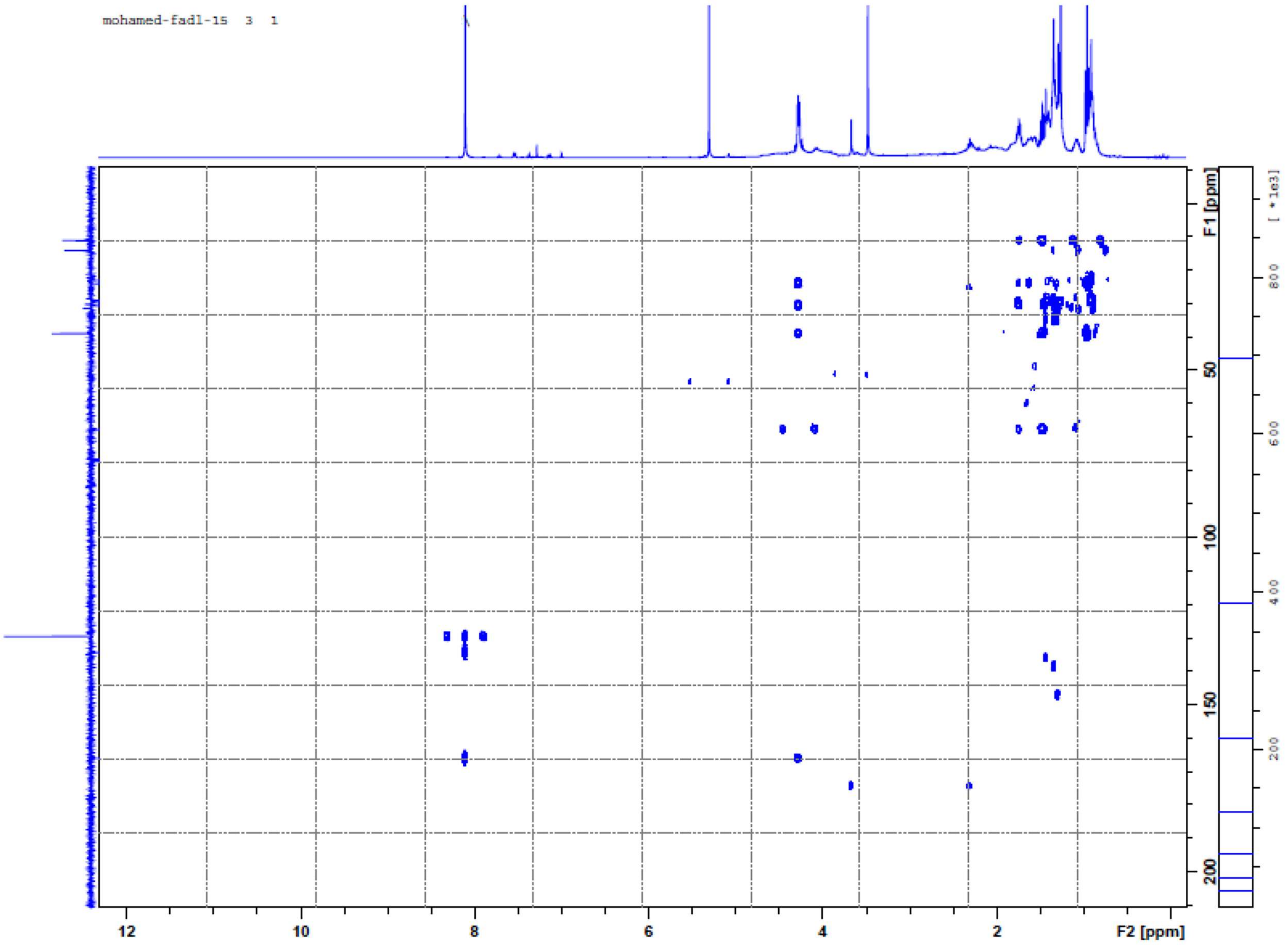

Fig. S7 HMBC-2 spectrum of the isolated compound

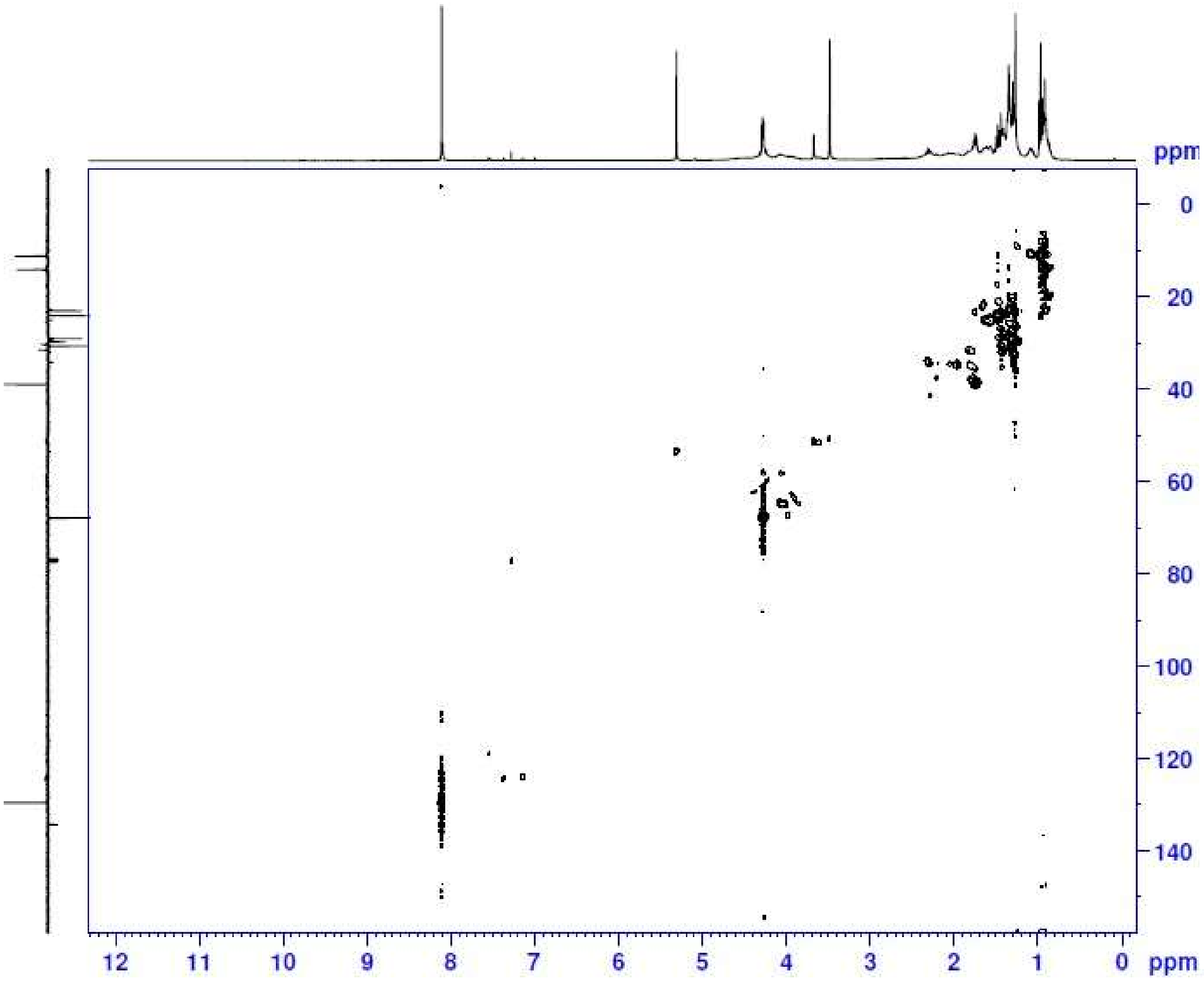

Fig. S8 HMQC-1 spectrum of the isolated compound

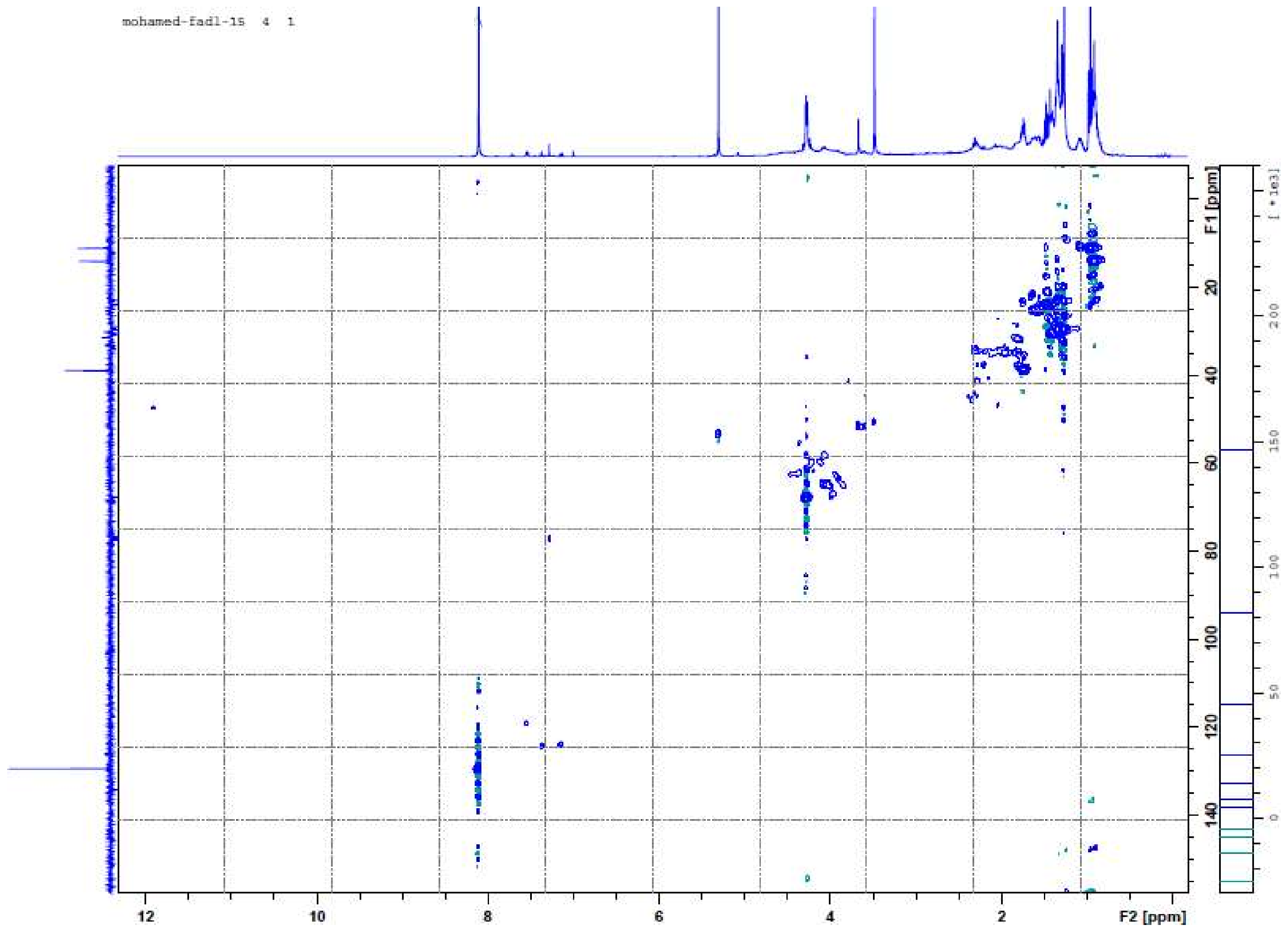

Fig. S9 HMQC-2 spectrum of the isolated compound

Center for DRUG DISCOVERY RESEARCH and DEVELOPMENT | Page 8
Openlynx Report -
Sample: 1313 | Vial:1:F,8 | ID:
Date:22-Jan-2024 | Time:15:42:11

Printed: Tue Jan 23 11:41:59 2024

Peak ID	Time	Error PPM
6	25.72	

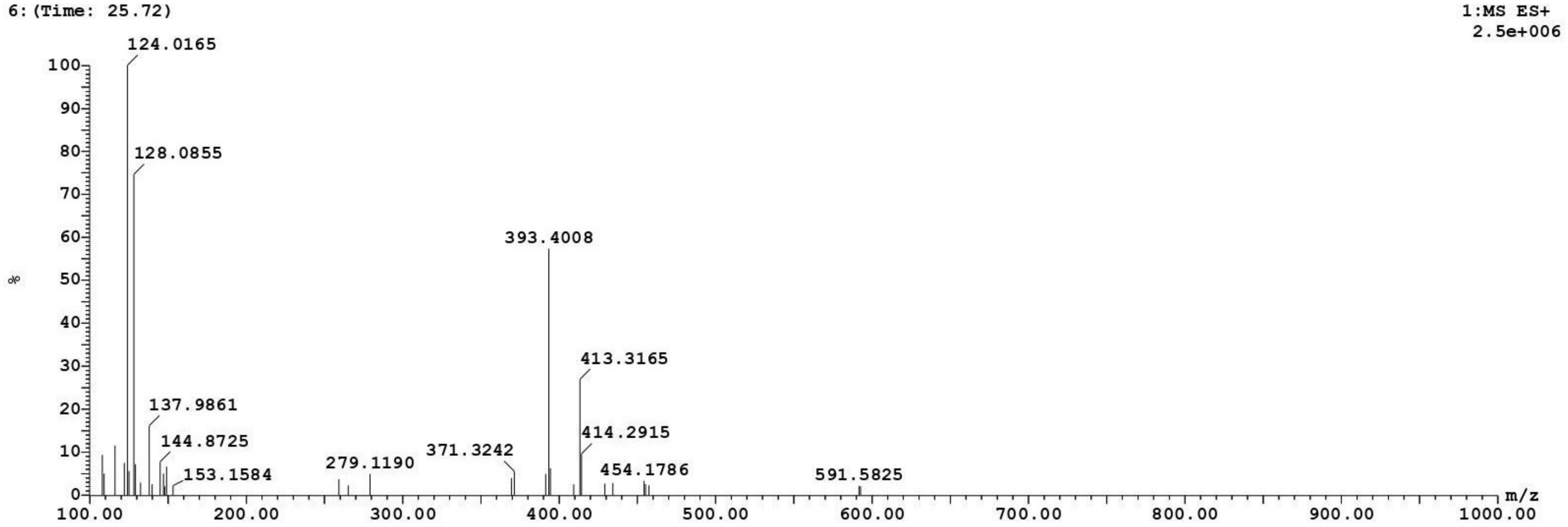

Fig. S10 (+) ESI-MS spectrum of the isolated compound

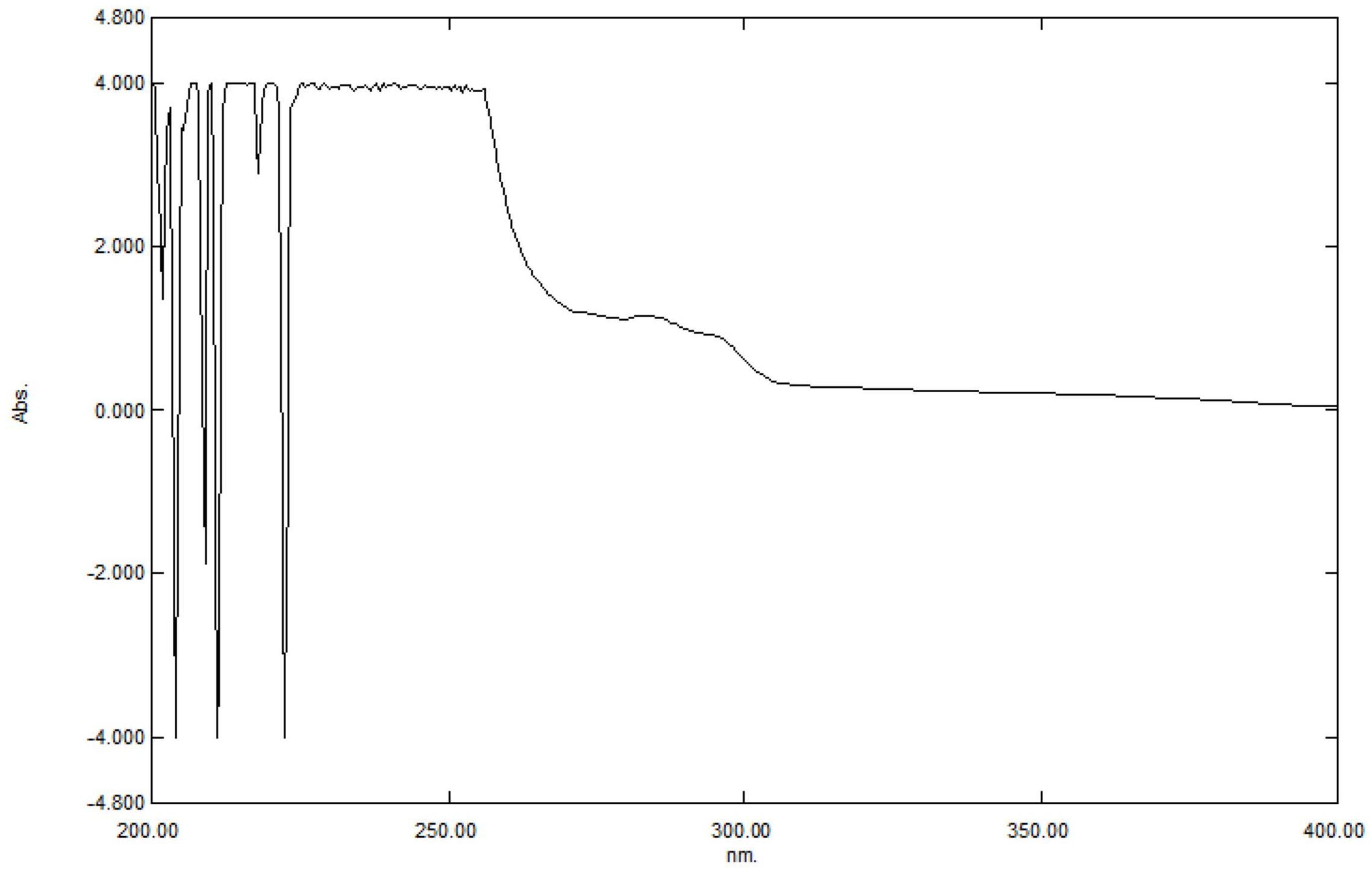

Fig. S11 Absorbance of UV by the isolated compound

Raman results

Band position (cm^{-1})	Assignments
3081	Ph-H stretching
2935	C-H of CH3 stretching
2877	C-H of CH2 stretching
1725	C=O stretching
1615	Ring quadrant stretch
1445	C-H bending of CH
1383	COO symmetric stretching , C-H bending of CH3,
1278	C-O stretching
1199	sym. stretching of para-substituted benzene
1172	In plane C-H bending (ring)
1113-1092	C-C stretching
871	C-O-C, C-H of aromatic ring bending, C-C-O bending

PCR detection of genes related to bioactive metabolites biosynthesis

1- Nonribosomal peptide synthase (NRPS) gene

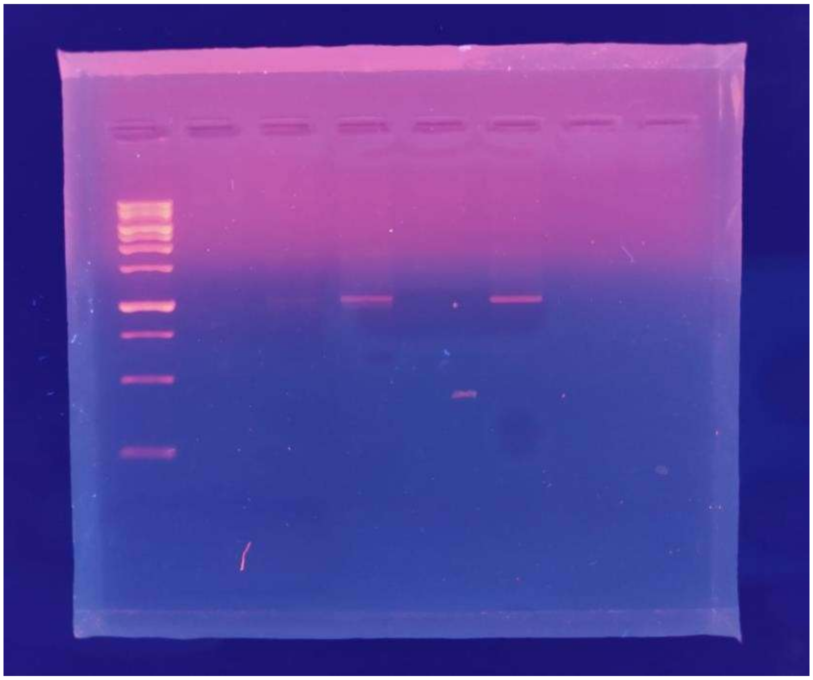

2- Malonyl-CoA transacylase *ItuD* lipopeptide gene

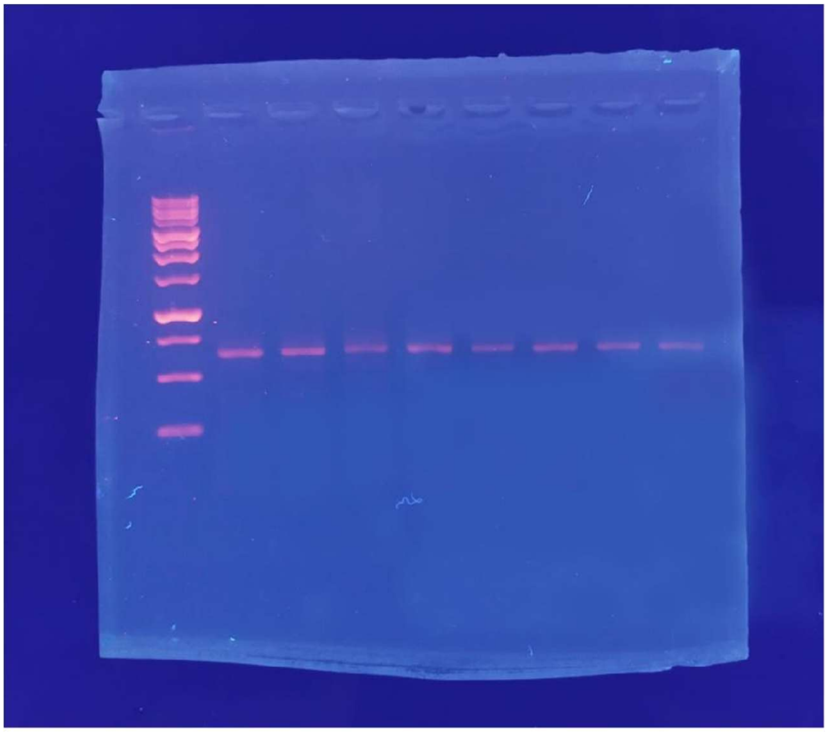

BLAST Results

Table 1. Sequence alignment of NRPS A domains from a Rhizosphere Strain, *Sphingomonas sanguinis* DM with those submitted to GenBank.

Description	Scientific Name	Max Score	Total Score	Query Cover	E value	Per. Ident	Acc. Len	Accession
surfactin non-ribosomal peptide synthetase SrfAA [Bacillus subtilis]	Bacillus subtilis	250	250	99%	4e-73	66.15%	3587	WP_258994967.1
surfactin non-ribosomal peptide synthetase SrfAA [Bacillus subtilis]	Bacillus subtilis	250	250	99%	5e-73	66.15%	3587	WP_129133870.1
surfactin non-ribosomal peptide synthetase SrfAA [Bacillus subtilis]	Bacillus subtilis	250	250	99%	5e-73	66.15%	3587	WP_121591425.1
surfactin non-ribosomal peptide synthetase SrfAA [Bacillus subtilis]	Bacillus subtilis	249	249	99%	1e-72	65.64%	3587	WP_103330354.1
surfactin non-ribosomal peptide synthetase SrfAA [Bacillus subtilis]	Bacillus subtilis	249	249	99%	2e-72	66.15%	2603	WP_163117763.1
surfactin non-ribosomal peptide synthetase SrfAA [Bacillus subtilis]	Bacillus subtilis	249	249	99%	2e-72	66.15%	3587	WP_277710175.1
surfactin non-ribosomal peptide synthetase SrfAA [Bacillus subtilis]	Bacillus subtilis	248	248	99%	3e-72	66.15%	2537	WP_095252716.1
surfactin non-ribosomal peptide synthetase SrfAA [Bacillus subtilis]	Bacillus subtilis	248	248	99%	3e-72	66.15%	2645	WP_283857660.1
non-ribosomal peptide synthetase [Bacillus subtilis]	Bacillus subtilis	248	248	99%	3e-72	66.15%	3587	WP_227058517.1
surfactin non-ribosomal peptide synthetase SrfAA [Bacillus subtilis]	Bacillus subtilis	248	248	99%	3e-72	65.13%	3587	WP_260665508.1
surfactin non-ribosomal peptide synthetase SrfAA [Bacillus subtilis]	Bacillus subtilis	248	248	99%	3e-72	66.15%	2517	WP_077671227.1
surfactin non-ribosomal peptide synthetase SrfAA [Bacillus subtilis]	Bacillus subtilis	248	248	99%	3e-72	66.15%	2594	WP_124048528.1
surfactin non-ribosomal peptide synthetase SrfAA [Bacillus subtilis]	Bacillus subtilis	248	248	99%	3e-72	65.13%	3587	WP_088272162.1
non-ribosomal peptide synthetase [Bacillus subtilis]	Bacillus subtilis	248	248	99%	3e-72	66.15%	3587	WP_223497600.1
non-ribosomal peptide synthetase [Bacillus subtilis]	Bacillus subtilis	248	248	99%	3e-72	66.15%	3587	WP_213419535.1
surfactin non-ribosomal peptide synthetase SrfAA [Bacillus subtilis]	Bacillus subtilis	248	248	99%	4e-72	66.15%	3587	WP_286317446.1
surfactin non-ribosomal peptide synthetase SrfAA [Bacillus subtilis]	Bacillus subtilis	248	248	99%	4e-72	66.15%	2603	WP_165634642.1

Table 2. BLAST results exhibit the percentage similarity between the lipopeptide *ItuD* fragments from *Sphingomonas sanguinis* DM and other related proteins available in GenBank database.

Description	Scientific Name	Max Score	Total Score	Query Cover	E value	Per. Ident	Acc. Len	Accession
bacillomycin D biosynthesis malonyl-CoA transacylase BamD [Bacillus amyloliquefaciens]	Bacillus amyloliquefaciens	86.7	145	51%	1e-25	93.02%	400	WP_101670426.1
bacillomycin D biosynthesis malonyl-CoA transacylase BamD [Bacillus velezensis]	Bacillus velezensis	89.0	145	51%	1e-25	95.35%	400	WP_254003784.1
bacillomycin D biosynthesis malonyl-CoA transacylase BamD [Bacillus amyloliquefaciens gro...	Bacillus amyloliquefaciens group	89.0	145	47%	1e-25	95.35%	400	WP_015239954.1
bacillomycin D biosynthesis malonyl-CoA transacylase BamD [Bacillus velezensis]	Bacillus velezensis	89.0	145	47%	1e-25	95.35%	400	WP_095284077.1
bacillomycin D biosynthesis malonyl-CoA transacylase BamD [Bacillus]	Bacillus	89.0	145	47%	1e-25	95.35%	400	WP_015417573.1
bacillomycin D biosynthesis malonyl-CoA transacylase BamD [Bacillus sp. JNUCC-22]	Bacillus sp. JNUCC-22	89.0	145	47%	1e-25	95.35%	400	WP_216062299.1
bacillomycin D biosynthesis malonyl-CoA transacylase BamD [Bacillus sp. AF12]	Bacillus sp. AF12	89.0	145	47%	1e-25	95.35%	400	WP_229041382.1
bacillomycin D biosynthesis malonyl-CoA transacylase BamD [Bacillus]	Bacillus	89.0	145	47%	1e-25	95.35%	400	WP_077391888.1
bacillomycin D biosynthesis malonyl-CoA transacylase BamD [Bacillus amyloliquefaciens]	Bacillus amyloliquefaciens	89.0	145	47%	1e-25	95.35%	400	WP_222836065.1
bacillomycin D biosynthesis malonyl-CoA transacylase BamD [Bacillus velezensis]	Bacillus velezensis	88.6	144	47%	1e-25	95.35%	400	WP_220007288.1
bacillomycin D biosynthesis malonyl-CoA transacylase BamD [Bacillus velezensis]	Bacillus velezensis	88.6	144	47%	2e-25	95.35%	400	WP_059367372.1
bacillomycin D biosynthesis malonyl-CoA transacylase BamD [Bacillus velezensis]	Bacillus velezensis	88.6	144	47%	2e-25	95.35%	400	WP_121984453.1
bacillomycin D biosynthesis malonyl-CoA transacylase BamD [Bacillus amyloliquefaciens gro...	Bacillus amyloliquefaciens group	88.6	144	47%	2e-25	95.35%	400	WP_257726799.1
bacillomycin D biosynthesis malonyl-CoA transacylase BamD [Bacillus velezensis]	Bacillus velezensis	88.6	144	51%	2e-25	95.35%	400	WP_263612167.1
bacillomycin D biosynthesis malonyl-CoA transacylase BamD [Bacillus amyloliquefaciens gro...	Bacillus amyloliquefaciens group	88.6	144	47%	2e-25	95.35%	400	WP_052827501.1
bacillomycin D biosynthesis malonyl-CoA transacylase BamD [Bacillus velezensis]	Bacillus velezensis	88.6	144	47%	2e-25	95.35%	400	WP_064107341.1
bacillomycin D biosynthesis malonyl-CoA transacylase BamD [Bacillus velezensis]	Bacillus velezensis	88.6	144	47%	2e-25	95.35%	400	WP_136396410.1
